# Contextual Analysis and Implementation Strategies for an Age‐Friendly Emergency Department Uptake: The FRED Study Protocol

**DOI:** 10.1111/jgs.70230

**Published:** 2025-12-02

**Authors:** Alisa Cantarero Fernandez, Christian H. Nickel, Thomas Dreher‐Hummel, Florian Grossmann, Luca Ünlü, Christopher R. Carpenter, Pieter Heeren, Robert A. C. Ruiter, Michael Simon, Franziska Zúñiga

**Affiliations:** ^1^ Nursing Science, Department Public Health University of Basel Basel Switzerland; ^2^ Department of Emergency Medicine University Hospital of Basel Basel Switzerland; ^3^ Faculty of Medicine University of Basel Basel Switzerland; ^4^ Department of Acute Medicine University Hospital of Basel Basel Switzerland; ^5^ Department of Emergency Medicine Mayo Medical Center Rochester Minnesota USA; ^6^ Faculty of Medicine and Life Sciences Hasselt University Hasselt Belgium; ^7^ Department of Public Health and Primary Care KU Leuven Leuven Belgium; ^8^ Department of Work and Social Psychology Maastricht University Maastricht the Netherlands

**Keywords:** emergency department, frailty, geriatric emergency medicine, implementation science, older adults

## Abstract

**Background:**

Older adults frequently present to the Emergency Department (ED). In response, a Swiss university hospital introduced age‐friendly interventions and achieved Geriatric Emergency Department Accreditation (GEDA) by the American College of Emergency Physicians (ACEP). However, the impact of previously introduced interventions and the reasons behind emergency clinicians' varying uptake or lack of continued use remain unclear. To further improve patient outcomes, conducting a contextual analysis to identify implementation barriers and facilitators is crucial, followed by the development of tailored implementation strategies supporting the sustainable uptake of all age‐friendly program elements. The project's overall aim is to systematically promote the uptake and sustainable re‐implementation of the existing age‐friendly ED program. The first study phase outlined in this protocol (“Phase A”) focuses on 2 key objectives: (1) to assess current age‐friendly interventions in the ED and identify barriers and facilitators affecting their reach, adoption, implementation, and maintenance; (2) to develop tailored implementation strategies for re‐implementing program elements.

**Methods:**

This project uses a modified implementation mapping in 5 Steps across 2 Phases. Phase A includes Steps 1–4: (1) conducting a contextual analysis using a mixed‐methods design combining observations, interviews, patient chart reviews, E‐survey and a Gemba walk; (2) identifying expected intervention and implementation outcomes, performance objectives; (3) adapting, extending, or developing tailored implementation strategies based on the Expert Recommendations for Implementing Change taxonomy; and (4) co‐designing an implementation protocol to guide re‐implementation. The follow‐up Phase B will involve the re‐implementation of the intervention elements and co‐designing the evaluation protocol (Step 5) for the implementation process.

**Conclusion:**

Age‐friendly EDs are essential for person‐centered emergency care, enhancing safety and quality of care for older adults. This study will provide insights into adaptable, evidence‐informed implementation strategies that support behavioral change among emergency clinicians to increase patient reach and sustainability of age‐friendly interventions for complex ED settings.

## Summary

- Key points
  ○ Widespread implementation of age‐friendly ED programs remains limited due to an incomplete understanding of context‐specific barriers and facilitators.
  ○ There is a need for tailored implementation strategies that support sustainable change in a fast‐moving ED context.
  ○ Fostering the sustainment of an age‐friendly ED will go hand in hand with enhancing geriatric expertise by empowering the workforce to provide person‐centered care and improve health outcomes.
- Why does this paper matter?
  ○ The FRED project is a systematic re‐implementation of an age‐friendly emergency department program that leverages implementation science.
  ○ The primary objective of this effort is to further improve and sustain the quality of care for older patients by increasing emergency clinician adoption and patient reach of age‐friendly interventions, while enhancing person‐centeredness.
  ○ The proposed multi‐framework approach and results of the FRED project can provide sustainable, evidence‐based solutions that are adaptable to different emergency settings.

## Graphical abstract

The age‐friendly Emergency Department (ED) program. This graphical abstract illustrates how the FRED (age‐FRiendly Emergency Department) project structures strengthen an age‐friendly ED by linking key Geriatric ED Accreditation (GEDA) requirements—Workforce, Operational Structure, and Infrastructure—with the Geriatrics 5Ms Framework to further advance sustainable, person‐centered care for older adults.

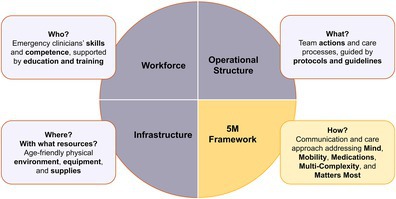

## 1 Introduction

Older adults aged 65 years and older are presenting to Emergency Departments (EDs) worldwide with increasing frequency [1, 2]. Age‐friendly ED programs promote a comprehensive approach and can potentially reduce admission rates and costs [3, 4, 5, 6]. However, their scalable implementation and sustainability are often challenging since a systematic approach to understand the context and develop implementation strategies is lacking.

**FIGURE 1:**
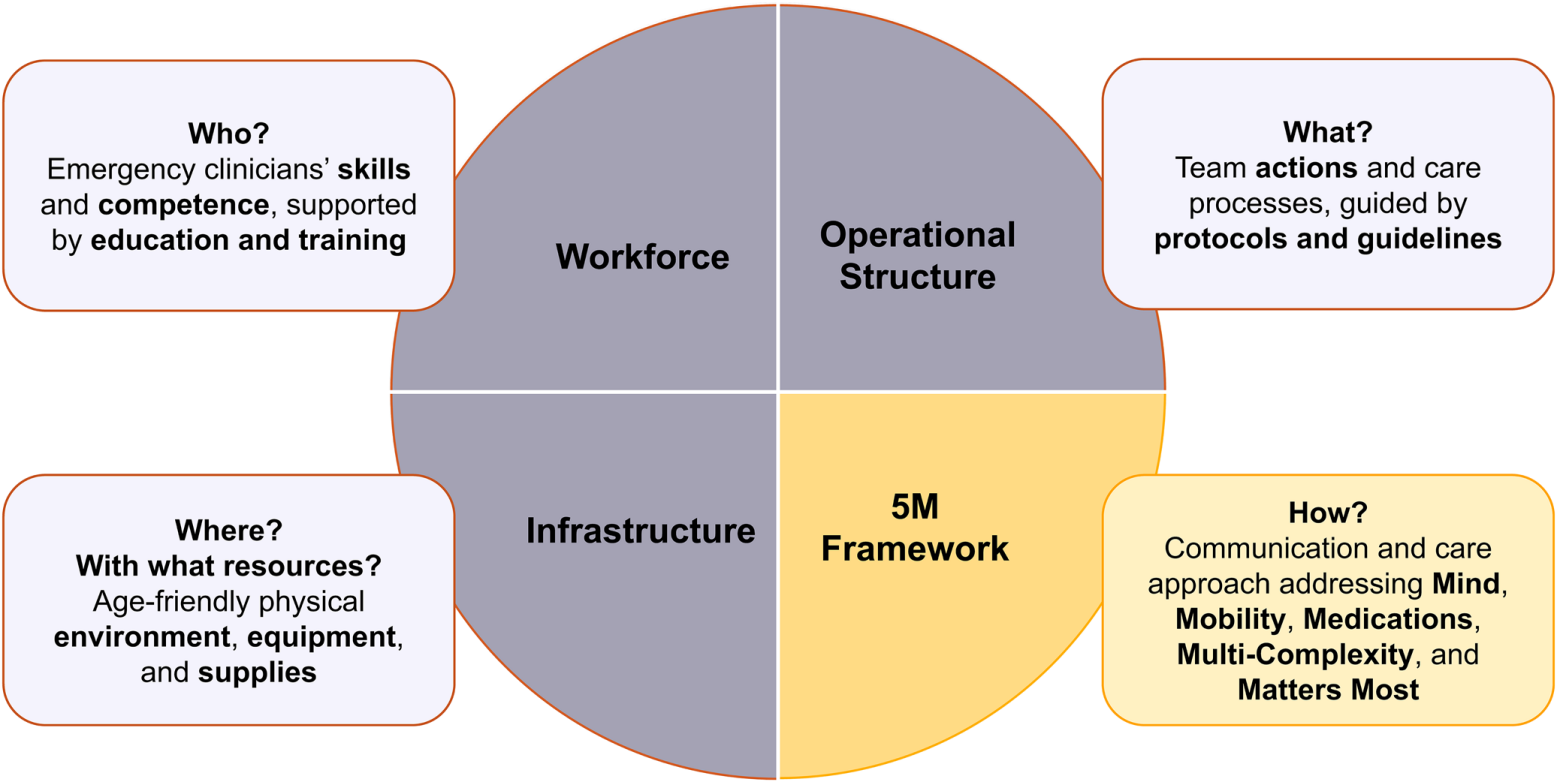
The complex intervention was developed within the FRED (age‐FRiendly Emergency Department) project. The age‐friendly Emergency Department (ED) program builds on existing intervention elements within the targeted ED setting and is now systematically organized and guided by the Geriatric Emergency Department Accreditation (GEDA) components—Workforce, Operational Structure, and Infrastructure—and aligned with the Geriatrics 5Ms Framework—Mind, Mobility, Medications, Multi‐Complexity, and Matters Most [10, 12, 13, 14]. Together, these components illustrate who provides care, what actions and processes are used, where care takes place, and how age‐friendly principles support ongoing improvements for older adults.

In 2018, the Geriatric Emergency Department Accreditation (GEDA) program was launched by the American College of Emergency Physicians (ACEP) [7, 8, 9]. GEDA aims to improve care for older patients by defining key components of a geriatric ED, aligning stakeholders, and tracking outcomes through a quality dashboard [7]. For example, Level 1 sites must implement 20 care processes and maintain a Quality Improvement plan with metrics tracking, while Level 3 sites require four care processes and no formal tracking [10]. In 2025, 575 EDs worldwide were GEDA‐accredited (31 Level 1, 75 Level 2, 469 Level 3), 95% in the United States. Accredited in 2024, our ED is the first Level 1 site outside the United States [11]. In our FRED project (age‐FRiendly ED), we have structured GEDA into 3 main components (workforce, operational structure, and infrastructure) and aligned this with the 5Ms framework (mind, mobility, medication, multi‐complexity, and what matters most) [10, 12, 13, 14]. Combined, they form a complex intervention to improve the clinical outcomes of older patients (see Figure 1) [12, 15].

**FIGURE 2:**
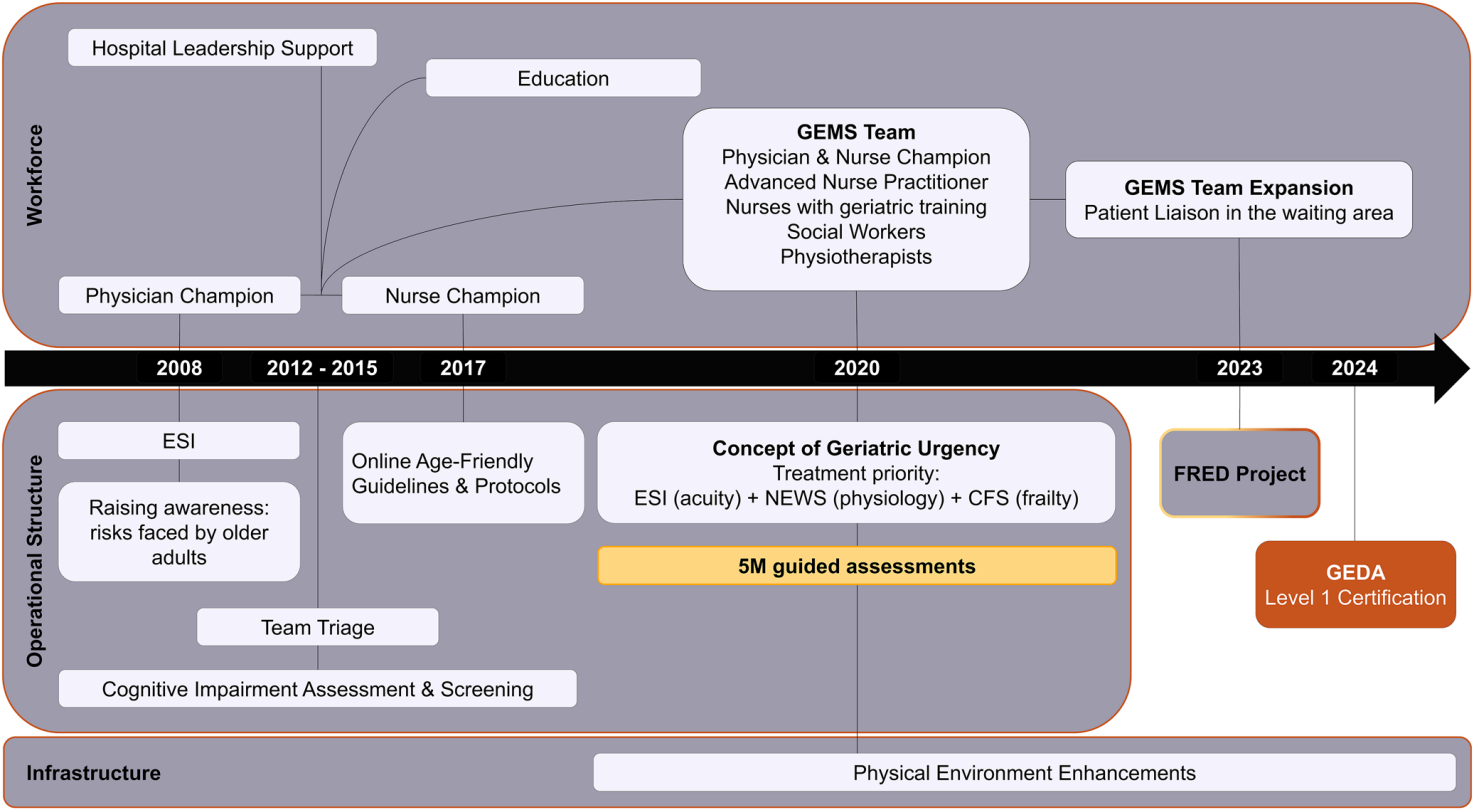
Overview of FRED's age‐friendly intervention elements implemented at the University Hospital Basel, Northwestern Switzerland (2008–2024). The timeline shows how existing age‐friendly practices evolved and were integrated into the age‐friendly ED program developed within the FRED project. In 2024, these efforts resulted in GEDA Level 1 accreditation, requiring verification of 20 care processes, quality improvement activities, and metrics tracking (see Supporting Information S1) [32]. 5 M, Mind, Mobility, Multi‐Complexity, Medications, Matters Most [14]; CFS, Clinical Frailty Scale [28, 29, 30]; ESI, Emergency Severity Index [33]; FRED, age‐FRiendly Emergency Department; GEDA, Geriatric Emergency Department Accreditation [10]; GEMS, Geriatric Emergency Medicine Specialist; NEWS, National Early Warning Score [34].

In the United States, the Centers for Medicare & Medicaid Services (CMS) has introduced new age‐friendly hospital measures grounded in the 5 M framework [14], which promotes integrating key geriatric principles into ED practice and is supported by the ACEP [9, 16]. In Europe, the formation of the European Taskforce for Geriatric Emergency Medicine (ETGEM)—a collaboration between the geriatric section of the European Society for Emergency Medicine (EUSEM) and the Urgent Care section of the European Geriatric Medicine Society (EuGMS)—has developed a curriculum and research agenda for Geriatric Emergency Medicine, aligning with international standards for integration into practices [15, 17, 18]. Locally, the University Hospital Basel in Northwestern Switzerland has implemented various age‐friendly interventions since 2008: This involved raising awareness of the risks faced by older adults [19, 20, 21, 22], developing and validating a delirium screening tool [23, 24], introducing team triage for collective decision‐making to enhance triage quality in older patients [25, 26], implementing age‐friendly standards [27], validating the Clinical Frailty Scale (CFS) for the ED setting, creating and implementing a frailty‐adjusted risk stratification tool [28, 29, 30], and establishing a Geriatric Emergency Medicine Specialist (GEMS) team. These age‐friendly initiatives aim to improve care for older adults by promoting person‐centered care, which respects individuals' rights, builds trust, and focuses on what matters most to patients [13, 14, 31]. Figure 2 summarizes our interventions over the last 15 years. For detailed information about the foundations and motivation behind our implementation efforts, please see Supporting Information S1.

However, it is challenging to implement complex interventions in the ED setting due to non‐linear workflows, large patient volumes with frequent interruptions and high decision density, multiple and ever‐increasing time‐dependent emergency quality metrics, and high ED clinician attrition with subsequent replacement. Such pressures compromise the sustainability of implementation [15, 35, 36]. Consequently, age‐friendly assessments such as CFS assessment are not consistently applied to all older patients at ED presentation [37, 38]. In addition, the frequency and fidelity of assessments across GEDA EDs are not uniform [39].

While GEDA's program serves as a starting point for improving ED processes, its impact likely remains limited without an understanding of the reasons underlying clinicians' varying uptake [38, 40]. To further improve care, it is essential to (1) determine by whom, how, when, and why interventions are applied; (2) move beyond simply defining best practices to assess their value for patients; and (3) determine how these can be sustainably (re)implemented in the ED [41, 42]. This marks the starting point for the FRED project. In our context, “re‐implementation” refers to revisiting existing intervention elements that have already been introduced in practice, assessing their current uptake, and then systematically redesigning both intervention and implementation activities to support the interventions' reintroduction [43]. This process might include adapting, expanding, or even replacing intervention elements where indicated. Existing strategies for sustained implementation often focus on different combined intervention elements rather than the program as a whole, resulting in insufficient guidance on how to sustainably implement a comprehensive age‐friendly ED [44]. To measure the success of an age‐friendly ED, outcomes should reflect meaningful improvements in patient care. Instead of relying solely on traditional measures like 30‐day mortality, FRED focuses on innovative, person‐centered metrics [45, 46, 47]. Further, there is a need for structured, evidence‐informed methods to fit interventions to context, foster behavior change, and reach a sustained impact on patient outcomes [15, 18]. This is the first contextual analysis examining the implementation of an age‐friendly ED program.

**FIGURE 3:**
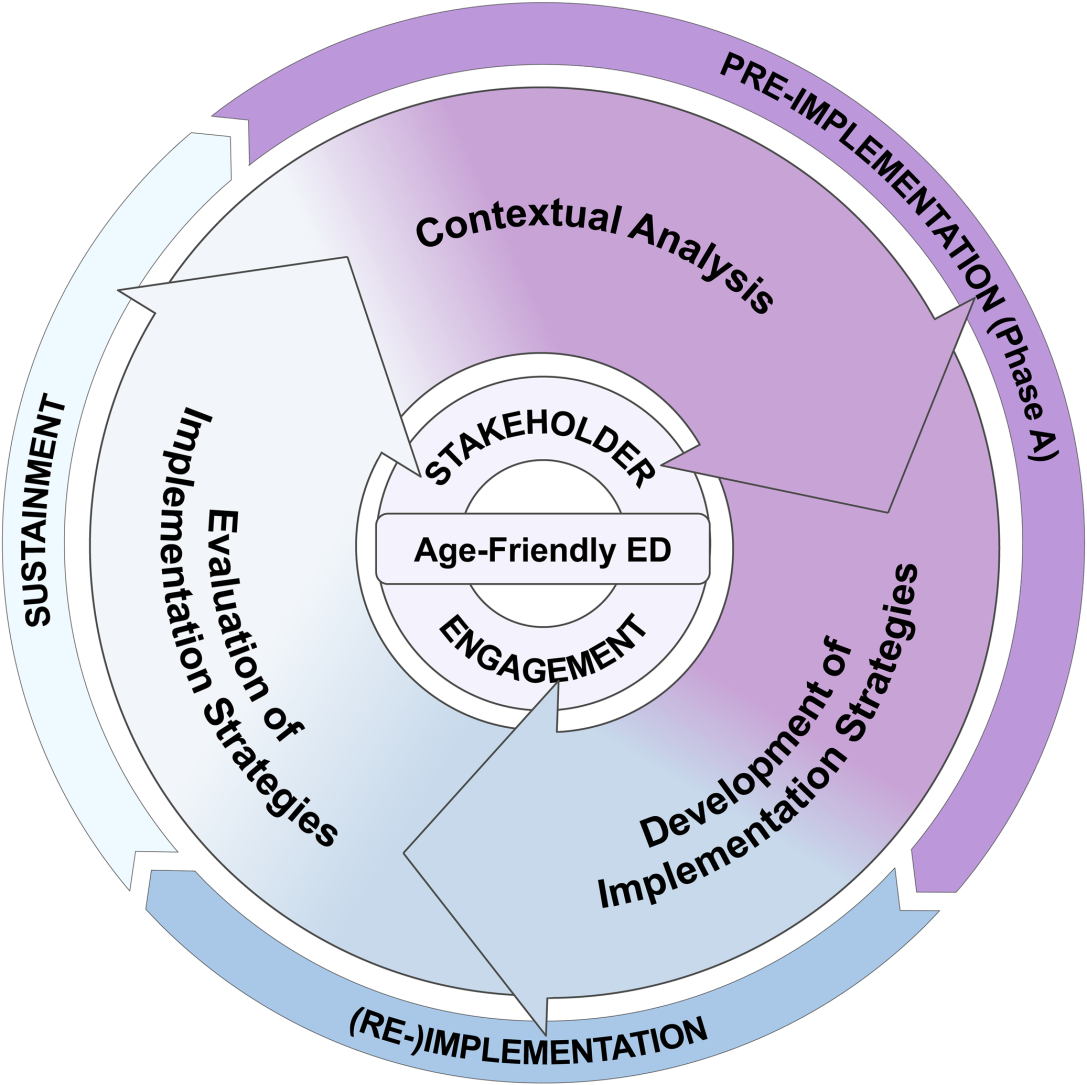
The multi‐framework approach of the FRED (age‐FRiendly Emergency Department) project shows how the program is built on implementation‐science principles [43]. The outer circle represents the implementation process, which moves from pre‐implementation (preparation) to (re‐)implementation (action) and sustainment (routine) of age‐friendly practices [50]. The inner circle outlines the core steps of this approach—contextual analysis, development of implementation strategies, and evaluation of these strategies [43]. In the current study, Phase A focuses on the first two core steps, aimed at understanding the local context and developing tailored strategies for implementation. Together, this multi‐framework approach forms a continuous learning system that promotes ongoing, age‐friendly improvements in quality of care. Phase B, which involves (re‐)implementation and sustainment, is outside the scope of this protocol.

We use an implementation science approach to ensure the sustainable uptake of GEDA's age‐friendly ED program [48]. Successful implementation depends on the effective use of implementation strategies to achieve implementation outcomes like reach (the number of older patients receiving interventions), adoption (clinicians' intent to integrate interventions), implementation (delivery of the intervention as intended and adaptations made during the process), and maintenance (sustained integration into daily routines) [49]. Implementation strategies, such as training, materials, coaching, or adaptation of processes, need to be tailored to the specific context to effectively overcome recognized barriers. For a structured development of fitting strategies, we use the modified 5‐step implementation mapping approach of Schultes et al. [43], which takes into account previous implementation efforts of existing interventions: (1) identify stakeholders and assess implementation barriers and facilitators; (2) assess intervention outcomes, implementation outcomes, and performance objectives; (3) assess (implicit) logic models and adapt implementation strategies; (4) co‐design implementation protocol; and (5) co‐design evaluation protocol [43]. For the overall project, we will group the 5 Steps into 2 Phases. Phase A (pre‐implementation) includes Steps 1 through 4. Step 5 is conceptually part of the pre‐implementation; however, it will be conducted in Phase B, which also includes the re‐implementation and sustainment stages and lies beyond the scope of this protocol (see Figure 3).

This study protocol aims to ensure transparency and reproducibility in ED‐based Geriatric Emergency Medicine implementation research. It serves as a potential guide for other EDs seeking to sustainably (re‐)implement age‐friendly ED programs. FRED's overall objective is to maximize the systematic uptake and sustainable re‐implementation of the age‐friendly ED program at one institution; this includes the critical evaluation of existing activities—supporting adaptation, expansion, or replacement of intervention elements through re‐implementation, and the removal of elements through de‐implementation where appropriate.

Phase A has 2 aims guided by the implementation mapping process: [43].

1. To assess the current implementation status of age‐friendly interventions in the ED and to identify barriers and facilitators that affect their successful reach, adoption, implementation, and maintenance.
2. To systematically map ED processes and develop tailored implementation strategies for re‐implementing elements of an age‐friendly ED program.

## 2 Methods

### 2.1 Context

#### 2.1.1 Setting and Overall Sample

The project is conducted at an urban tertiary care center in Switzerland. In 2024, about 56,000 patients were treated in the ED, and 1/3rd were aged 65 and older, with 40% of those living with at least mild frailty (CFS score of ≥ 5) [1]. The age threshold is based on factors like pension and retirement systems, demographic and statistical standards (Eurostat, WHO, UN) [51]. A total of 201 emergency clinicians, including 69 physicians (38 residents, 31 attending and triage liaison physicians), 135 nurses (1 Advanced Practice Nurse (APN), 124 registered nurses), and 20 medical support staff, are employed in the ED, and all are involved in age‐friendly interventions. The term ‘emergency clinicians’ refers to all ED healthcare professionals involved in emergency care.

#### 2.1.2 Age‐Friendly Triage Process and Workflows

**FIGURE 4:**
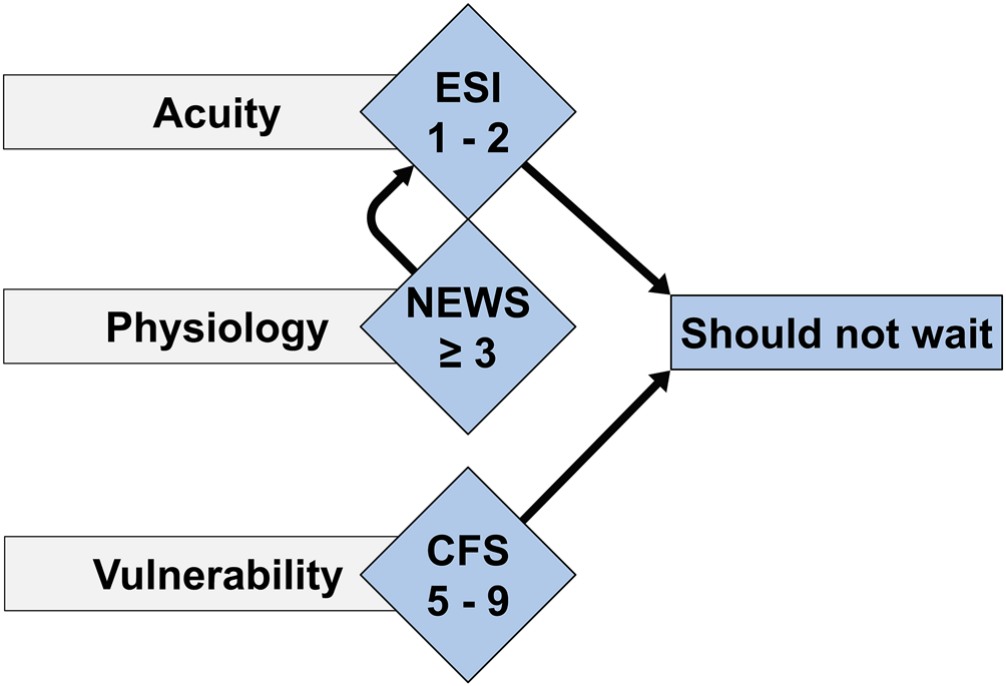
The University Hospital Basel's age‐friendly triage process consists of 3 components: Acuity (as assessed with a triage tool, ESI) [33], physiology (as determined by an aggregated vital sign score, NEWS) [34], and frailty (CFS) [28, 29, 30, 52]. Each component can escalate a patient's status to ‘should not wait’. Patients deemed stable (ESI of ≥ 3, NEWS of < 3) but living with frailty (CFS score of ≥ 5) will be streamed to an age‐friendly area, where tailored interventions occur. This approach ensures the integration of the special care needs of older patients into the ED workflow while remaining adaptable to individual patient needs. CFS, Clinical Frailty Scale; ESI, Emergency Severity Index; NEWS, New Early Warning Score.

We believe that older adults living with frailty should not experience delays and must be identified immediately upon ED arrival to facilitate timely risk assessment and the initiation of age‐friendly care processes. As with all patients, the Emergency Severity Index (ESI) is used to assess acuity by identifying patients who should not wait (ESI < 3) [33]. During triage, a complete set of vital signs is recorded to determine an aggregated vital signs score, an adapted version of the National Early Warning Score (NEWS) [34]. To identify vulnerability, we have integrated the CFS into the triage process [28, 30, 52]. A CFS score of ≥ 5 triggers timely treatment and allows early identification of patients who may benefit from elements of Comprehensive Geriatric Assessment (CGA) [15, 28, 29, 30]. To deliver the right treatment to the right patient at the right time and place with appropriate resources [53], triage clinicians consider geriatric urgency: frail older patients should not wait [28]. Additionally, older patients who are deemed stable but live with frailty (determined by an ESI of ≥ 3, NEWS of < 3, and CFS score of ≥ 5) are streamed directly to an age‐friendly area in our ED observation unit (see Figure 4).

Our multidisciplinary and interprofessional team additionally includes the GEMS team. The team comprises 1 physician champion, 1 nurse champion (APN), 12 nurses with at least a Bachelor's degree with geriatric training, social workers, physiotherapists, and a patient liaison in the waiting area. The GEMS team collaborates closely with emergency clinicians, other healthcare professionals, patients' formal and informal care partners, as well as community‐based services. The GEMS team operates on weekdays from 8 a.m. to 5 p.m.

If frailty is identified (CFS score of ≥ 5), the GEMS conducts older adults' assessments, guided by the 5 M Framework [12, 14, 17]. The results of the assessments help address individual needs, resulting in tailored interventions, personalized treatment plans, and a joint decision regarding disposition. Additionally, GEMS coordinates care with outpatient services or referrals to geriatric hospitals nearby. If patients are admitted, CFS scores are sent to the in‐house geriatric consultation service to facilitate further care coordination. Emergency clinicians continue core age‐friendly practices, such as screening for cognitive impairment, when the GEMS team is not on‐site.

### 2.2 Step 1: Identify Stakeholders and Assess Implementation Barriers and Facilitators

**FIGURE 5:**
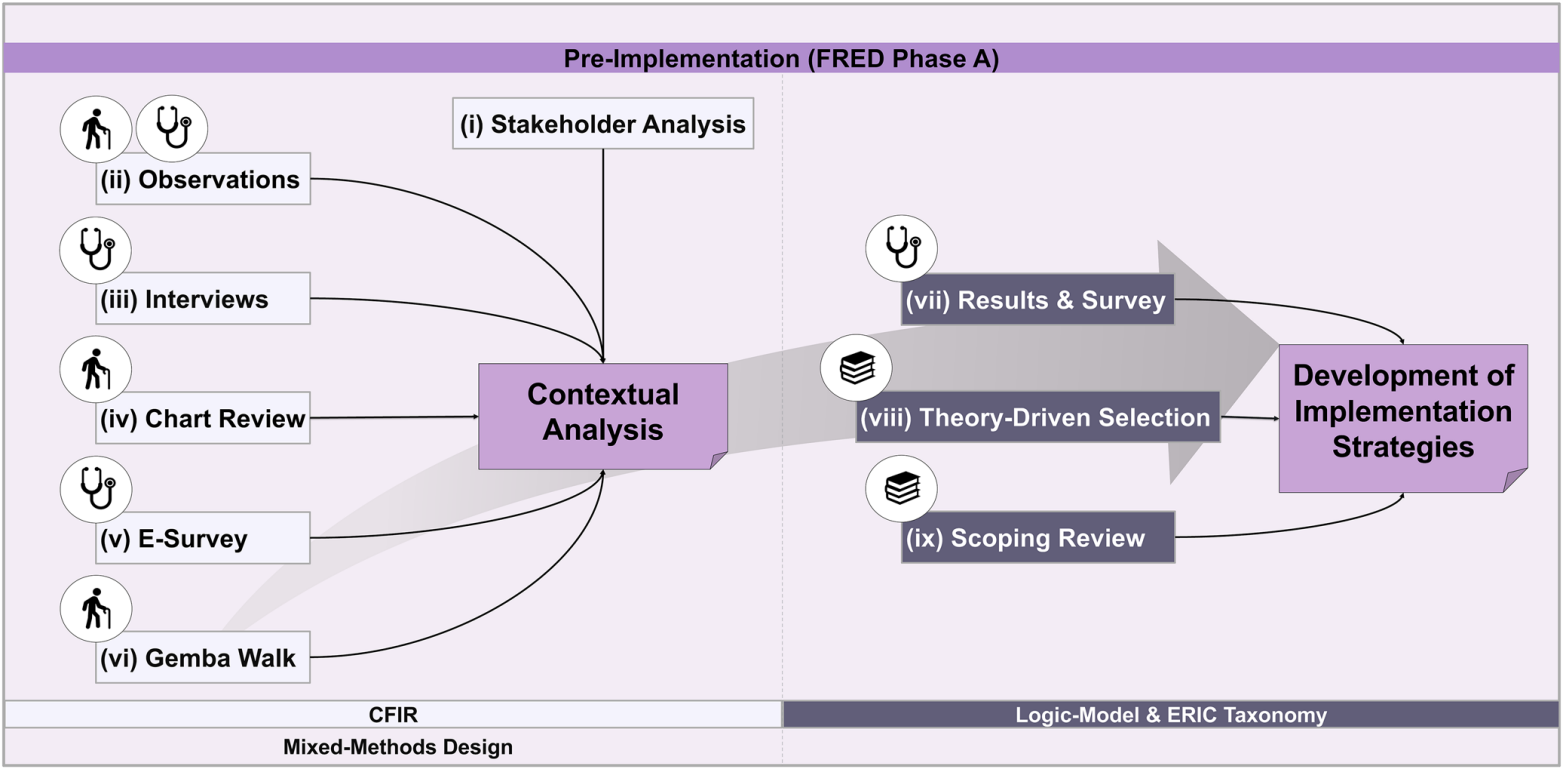
The methodological approach for Phase A (pre‐implementation) of this protocol. This figure illustrates the main elements of the contextual analysis (steps i–vi, see description in text) and the development of implementation strategies (steps vii–ix) carried out during the preparation phase. Both steps are supported by established theories, models, and frameworks, which help to ensure that the planned strategies are evidence‐based and tailored to the local emergency department context. CFIR, Consolidation Framework for Implementation Research [54]; ERIC, Expert Recommendations for Implementing Change [55].

In Step 1 of implementation mapping, we will use a convergent parallel mixed‐method study design to ensure an in‐depth understanding of contextual factors (see Figure 5) [56]. The contextual analysis has received an exemption from the Ethics Committee Northwest and Central Switzerland (Req‐2023‐01542).

#### 2.2.1 Stakeholder Analysis (i)

Using a co‐design approach, we will discuss each step with the stakeholder group in view of the guiding questions, data analysis, and interpretation of the results [57]. We identified our stakeholder group based on the 7 principal steps of stakeholder analysis: defining, long listing, mapping, visualizing, verifying, mobilizing, and evaluating [58]. This stakeholder group focuses on the individual level (patients, their informal care partners, and emergency clinicians) and organizational level (institutional Patient Advisory Board, management representatives, and researchers), sharing insights, experiences, and expertise. Although we are actively exploring system‐level connections beyond the FRED project scope, we did not include broader societal stakeholders due to limited resources. However, cross‐institutional positions and regular case discussions with the Department of Geriatric Medicine improve referral processes and continuity of care across institutional boundaries [59]. In the future, we plan to share FRED findings with community and system‐level stakeholders to strengthen regional collaboration and support broader institutional improvements.

#### 2.2.2 Observations and Informal Conversations (ii)

##### 2.2.2.1 Sample and Sampling

To assess the adoption and person‐centeredness of the existing age‐friendly ED interventions, the purposeful sampling strategy includes 12–18 emergency clinicians and 8–10 patients who must provide verbal consent or opt out actively. Eligibility criteria focus on heterogeneity among emergency clinicians (profession, experience in years, roles, age, sex) and patients (age ≥ 65, sex, ethnicity, frailty). Exclusions apply to those who verbally object, are disturbed by observation, or are in critical health situations.

##### 2.2.2.2 Data Collection

Starting in February 2024, currently ongoing, a researcher (ACF) and a research assistant visit the ED for half‐day periods to observe different shifts, all days of the week, and seasons. Observations cover process pathways (from ED presentation to discharge), perspectives of emergency clinicians and patients, and informal conversations. Observations and informal conversations are documented via structured field notes. Observations are guided by questions regarding how elements of the age‐friendly ED are adopted and maintained by emergency clinicians, as well as how person‐centeredness is delivered by emergency clinicians and experienced by patients and informal care partners in the ED. Informal conversations will help us understand certain observed sequences, actions, and reactions of the person who was observed.

##### 2.2.2.3 Data Analysis

The thematic analysis will follow a hybrid approach, combining deductive and inductive strategies in an iterative process. The analysis will be structured around the three predefined GEDA domains (workforce, operational structure, and infrastructure) as deductive parent categories. Within these domains, inductive coding will be employed to identify emerging themes. The coding and theme development process will be refined through regular team discussions and exchanges, ensuring consistency and rigor among the research group, including data collectors [60, 61].

#### 2.2.3 Interviews (iii)

##### 2.2.3.1 Sample and Sampling

The purposeful sampling strategy includes 5–10 emergency clinicians, including physicians, nurses, and ED leadership, as well as the hospital's executive leadership team, who will provide written informed consent. Eligibility criteria focus on heterogeneity among emergency clinicians (e.g., opinion leaders, part‐ or full‐time workers). Excluded are stakeholders outside the hospital setting (e.g., community services, primary care), as the scope of this study focuses on those directly involved in the ED environment and hospital‐level decision‐making.

##### 2.2.3.2 Data Collection

Guided by the results of the observations, semi‐structured interviews will be conducted in a separate room in the ED and audio‐recorded, lasting up to 30 min each, in autumn 2025.

##### 2.2.3.3 Data Analysis

The thematic analysis will follow the same approach as with the observation data [60, 61]. Afterward, themes from the interviews and observation/informal conversations will be merged.

#### 2.2.4 Routine Health Data (iv)

##### 2.2.4.1 Sample and Sampling

To assess the reach of existing age‐friendly ED interventions, we use data from the Electronic Health Records of all patients who visited the ED in February 2024 (1564 ED patient cases), both inpatients and outpatients aged 65 years and older. Excluded are patients with unknown identity and those treated in the resuscitation area.

##### 2.2.4.2 Data Collection

Before data collection, we consulted one attending physician, one resident physician, and two nurses to gather input on where age‐friendly ED interventions are documented, ensuring accurate and efficient data collection. All extracted data are entered into REDCap (Research Electronic Data Capture) without patient identifiers [62]. Three research assistants collect data independently. To ensure quality: (1) they undergo training using five random cases excluded from actual data collection; (2) attend weekly meetings with the responsible researcher to address any uncertainties; and (3) the responsible researcher checks their data entries for consistency by reviewing 30 randomly selected cases. Interrater reliability is assessed for the first 210 cases and each 20th case thereafter, while consensus cases are evaluated collaboratively. These methods are recommended in emergency medicine research, meeting 11 of 12 criteria (excluding hypothesis, as the data are descriptive) [63].

##### 2.2.4.3 Variables and Measurements

Data extraction covers 17 automatically extracted variables, including socio‐demographics, frailty, and patient flow data. Further 51 variables need manual extraction, covering intervention elements of an age‐friendly ED guided by GEDA's care process list [32]: (1) baseline care processes (e.g., urinary catheter use); (2) medication safety (e.g., pain management); (3) specialty consultation resources (e.g., geriatric psychiatry consultation); (4) ED screenings (e.g., cognitive impairment, fall risk); (5) transition of care (e.g., referrals to geriatric‐specific follow‐up clinic); and (6) hospital operations (e.g., quick transfers for patients with delirium) (see Table S1).

##### 2.2.4.4 Data Analysis

First, a missing data management plan will be created, followed by a descriptive analysis (*n* (%), mean, etc.) using the statistical software R v4.X [64], outlining how the intervention elements reach the target patient population.

#### 2.2.5 E‐Survey (v)

At a later stage, a sub‐study was added to FRED for an in‐depth assessment of determinants for delirium care. This includes selected variables from our routine health data review (see 2.3.3) in combination with an E‐survey of emergency clinicians regarding their knowledge of delirium and use of the mCAM‐ED screening and assessment tool.

##### 2.2.5.1 Sample and Sampling

An E‐survey will be conducted among emergency clinicians to explore their delirium screening and assessment practices. Completing and returning the survey to the research team will provide informed consent.

##### 2.2.5.2 Data Collection and Measurements

The survey will be distributed via email to all emergency clinicians through REDCap [62]. Two distinct but overlapping surveys are developed: one for emergency nurses, consisting of 28 items with nine unique questions, and one for emergency physicians, consisting of 29 items with 10 unique questions (see Table S2). The surveys are designed using the Consolidated Framework of Implementation Research (CFIR) Interview Guide, which aids in creating tailored surveys based on CFIR domains (i.e., intervention characteristics, outer setting, inner setting, individual characteristics, and process) [54].

##### 2.2.5.3 Data Analysis

Responses from nurses and physicians will be described with percentages using the statistical software R v4.X [64]. Statistical comparison between the two groups will be done using a *Z*‐test.

#### 2.2.6 Gemba Walk and Focus Group (vi)

The Gemba walk, led by the hospital's Patient‐Centered Management, aims to observe and improve the experience of older patients in the ED [65].

##### 2.2.6.1 Sample and Sampling

Six patient volunteers from the hospitals' Patient Advisory Board participated in a Gemba walk on May 11, 2023, followed by a focus group on August 22, 2023.

##### 2.2.6.2 Data Collection

During the Gemba walk, participants answered guiding questions about enhancing the ED's experience for older patients. All participants explored different pathways in two 40‐min sessions with emergency clinician assistance. In the follow‐up focus group, patient experiences were discussed using open‐ended questions, and responses were recorded in structured field notes.

##### 2.2.6.3 Data Analysis

Data were categorized by the hospital's Patient‐Centered Management and reviewed by the research group. Knowledge mapping summarized the focus group discussions, creating thematic blocks for further analysis.

#### 2.2.7 Mixed‐Method Analysis Plan

The observations and chart review data will be collected and analyzed simultaneously to facilitate comparing and integrating results [56]. Identified consistencies will enhance credibility, while discrepancies may provide further insights or prompt additional investigations. Results will be visually compared in tables or graphs and reported through a cohesive narrative [56]. The interviews, e‐survey, and Gemba walk will serve to enrich and contextualize findings from the observations and chart review by offering additional explanatory and stakeholder‐informed perspectives [56]. We will use the CFIR framework and its domains to group insights [54].

### 2.3 Step 2: Assess Intervention Outcomes, Implementation Outcomes, and Performance Objectives

Based on Step 1, we will be able to clarify the intended intervention and implementation outcomes so far and assess both the degree of success based on the results of Step 1 and the existing barriers and facilitators. With the stakeholder group, we foster a shared understanding of our outcomes and whether a refinement is needed for Phase B of FRED (e.g., strengthen person‐centeredness as an intervention outcome, degree of reach, adoption, implementation, maintenance for implementation outcomes). We adapt, refine or confirm performance objectives, that is, the tasks that need to be completed when applying age‐friendly ED interventions (see Section 2.4.2) [43].

### 2.4 Step 3: Assess (Implicit) Logic Models and Adapt Implementation Strategies

We will build a logic model to visualize how previously applied and ongoing implementation strategies (e.g., team‐based trainings, brief daily whiteboard teaching, monthly extended education sessions during shift change, interprofessional case discussions, and integration of assessments into the electronic workflow) are expected to support the uptake, reach and sustainability (e.g., the integration into daily workflows) of the age‐friendly ED intervention elements (cf. Figure 2 and Supporting Information S1). Additionally, it will serve as a baseline for considering new or adapted implementation strategies that might better fit our context [43].

The development of the implementation strategies will be guided by 3 inputs (cf. Figure 5):

#### 2.4.1 Contextual Analysis Results and Survey (vii)

In our contextual analysis, we will use the CFIR framework to group barriers and facilitators to the implementation of an age‐friendly ED [54]. Next, we will describe implementation strategies previously used and explore implicit theories of change (e.g., ED clinicians' theories about why implementation works) or explicit theories of change (i.e., those grounded in formal models or frameworks). Clarifying and reflecting on current theories of change will inform our logic model and lead to adapting, expanding, or replacing current implementation strategies to better fit our specific context and setting with its barriers and facilitators [43]. To describe the selected implementation strategies, we will use the Expert Recommendations for Implementing Change (ERIC) taxonomy—a catalog of recommended implementation strategies. We will apply the CFIR‐ERIC Matching Tool to find ERIC strategies suitable to address specific CFIR domains [55, 66]. We will also take into account recent developments in how implementation strategies are linked to determinants of behavior, such as the COM‐B model (Capability, Opportunity, Motivation–Behavior) [54, 67]. To ensure the approach is relevant to the clinical setting, emergency clinicians will review the pre‐selection of implementation strategies through an online survey, rating their importance and feasibility to guide resource use [55].

#### 2.4.2 Theory‐Driven Selection of Methods to Influence Determinants of Behavior, Enhancing the Implementation Process (viii)

To support change at both the organizational level (e.g., hospital policies) and individual level (e.g., behavior of emergency clinicians), we will use the explicit theories identified in Step 2 to select implementation strategies that match the identified barriers and facilitators to perform a task. For example, if the failure to assess the CFS during triage (the task) is caused by a lack of awareness about its importance (the barrier), we will choose an implementation strategy aimed at increasing awareness, guided by the COM‐B model as the explicit theory of change. The selected strategy would then aim to enhance the psychological capability and reflective motivation of emergency clinicians to assess CFS during triage [43, 54, 67]. We will create a matrix that links each task (i.e., performance objective) with its barrier to explain why interventions are performed or not. This will produce clear ‘change objectives,’ showing what needs to change, for example, awareness, knowledge, skills, or environment, to promote the desired behavior [43]. This process clarifies the theory of change behind each implementation strategy, leading to adaptations, expansion, or replacements of existing strategies.

#### 2.4.3 Scoping Review (ix)

We will conduct a scoping review to explore implementation strategies that support the uptake, re‐implementation, and maintenance of both simple and complex interventions in the ED context [68].

### 2.5 Step 4: Co‐Design Implementation Protocol

Co‐designing the implementation protocol with stakeholders ensures understanding and practical application. The protocol will include updated intervention elements, expected outcomes, the logic model, performance and change objectives, theories of change, selected strategies, and an implementation timeline [43, 69].

## 3 Discussion

The uptake of quality geriatric care for older patients is central to enhancing person‐centered care, influencing the patient's outcomes [15, 31]. Understanding and addressing the specific needs of older patients and what matters most to them is a key concern of age‐friendly EDs [13, 14, 15, 70].

In re‐implementing an age‐friendly ED program, it is essential to examine contextual factors [35, 71], as well as involve key stakeholders—such as patients and emergency clinicians—who provide valuable perspectives to the implementation process [43]. A collaborative approach will foster the translation of research into clinical practice. Ultimately, a comprehensive approach from an implementation science perspective enables us to develop targeted strategies for successful uptake and sustainability [43].

On the micro level, the direct beneficiaries of the systematic and sustainable establishment of an age‐friendly ED program are both patients and emergency clinicians [3, 71]. In addition to enhancing patient satisfaction, implementing standardized processes and evidence‐based guidelines results in more structured and potentially more efficient care, which, over time, leads to clearer procedures and can improve job satisfaction for emergency clinicians [3, 71]. At the meso level, the program may reduce hospital admissions, decrease adverse patient outcomes, and lower long‐term hospital costs [3, 71]. On the macro level, this project will contribute to research and innovation in geriatric emergency care. Using a structured approach with Implementation Mapping, we will provide a blueprint—grounded in contextual insights and implementation outcomes—that serves to inform and inspire similar efforts in other EDs globally. By sharing our lessons learned, we aim to enable more targeted, sustainable, and system‐sensitive adaptations across diverse ED settings.

## 4 Conclusion

The structured and sustainable re‐implementation of age‐friendly ED aims to further improve the care of older patients in the ED setting, meeting their specific needs, ensuring their safety, and incorporating what matters most to them. A structured implementation science approach has the potential for building sustainable changes to sustainably integrate the age‐friendly ED program in daily routine. Ultimately, re‐implementing an age‐friendly ED program will benefit our patients, informal care partners, emergency clinicians, and other healthcare providers on a broader scale.

## Author Contributions

The research group: Alisa Cantarero Fernandez, Christian H. Nickel, Thomas Dreher‐Hummel, Florian Grossmann, Luca Ünlü, Michael Simon, Franziska Zúñiga conceived the study, developed the study design, supervised, and contributed to data acquisition and analysis planning. The external experts: Christopher R. Carpenter, Pieter Heeren, Robert A.C. Ruiter were regularly consulted throughout the development of the protocol and provided input during all phases leading up to data collection. All authors contributed to manuscript revisions and approved the final version.

## Funding

This work was supported by the Nursing Science Foundation Switzerland (Grant Number: 3245‐2023). The funders had no role in the study's design, conduct, or reporting.

## Conflicts of Interest

Christian H. Nickel is a member of the geriatric sections of the European Society of Emergency Medicine and the International Federation of Emergency Medicine. He is also a member of the Geriatric Emergency Department Guidelines 2.0 writing group. He is an editor of medStandards, an online resource that provides evidence‐based emergency medicine protocols and no financial or personal conflicts to disclose. Christopher R. Carpenter is Associate Editor for the *Journal of the American Geriatrics Society*, leads the Society for Academic Emergency Medicine Guidelines for Reasonable and Appropriate Care in the Emergency Department Committee, serves on the American College of Emergency Physicians Clinical Policy Committee, is Chair of the American College of Emergency Physicians Geriatric Emergency Department Accreditation Advisory Board, and serves on the Clinician–Scientist Transdisciplinary Aging Research Leadership Core and no financial or personal conflicts to disclose. Pieter Heeren is a member of the geriatric section of the European Society of Emergency Medicine and no financial or personal conflicts to disclose. And other authors declare no conflicts to disclose.

## Supporting information


**Data S1:** Supporting Information.
