# Supplementary material for: Contextual Analysis and Implementation Strategies for an Age‐Friendly Emergency Department Uptake: The FRED Study Protocol
Source: J Am Geriatr Soc. 2025 Dec 2;74(2):365–76. doi: 10.1111/jgs.70230 (PMC12911551; doi:10.1111/jgs.70230)
Supplement: Supplementary file 1 — Data S1: Supporting Information. [file JGS-74-365-s001.pdf]

Supplementary Text 1: Foundations and Motivation behind our Implementation Efforts

In the U.S., the Centers for Medicare & Medicaid Services (CMS) has introduced new age-friendly hospital measures grounded in the 5M framework (mind, mobility, medication, multi-complexity, and what matters most),[1] which promotes integrating key geriatric principles into Emergency Department (ED) practice and is supported by the American College of Emergency Physicians (ACEP).[2–4]

In the European context, the formation of the European Taskforce for Geriatric Emergency Medicine (ETGEM)—a collaboration between the geriatric section of the European Society for Emergency Medicine (EUSEM) and the Urgent Care section of the European Geriatric Medicine Society (EuGMS)—has played a key role in bridging geriatric and emergency medicine.[5–7] These efforts are aligned with global initiatives, such as the minimum standards proposed by the International Federation for Emergency Medicine (IFEM).[8] ETGEM agreed that ED interventions for older adults should be pragmatic, feasible, and proportionate to the context.[7] ETGEM defined and validated a novel curriculum on Geriatric Emergency Medicine (GEM) designed to integrate geriatric and emergency medicine competencies. Domains and items for inclusion in the curriculum were derived through literature review and a nominal group workshop.[5] In addition, a European Research Agenda for GEM was created with the aim of providing an inventory and prioritization of future research questions. One of the top research priorities was to examine whether elements of Comprehensive Geriatric Assessment (CGA) would be beneficial for outcomes.[6] Based on the limited evidence available back then, expert clinical recommendations for the care of older people in EDs were provided.[7,9] Members of our FRED (age-FRiendly ED) research group, with leadership roles in ETGEM and contributions to the European GEM curriculum, have played a key role in integrating international and European policies into local ED practices.

Locally, the ED of the University Hospital Basel in Northwestern Switzerland has introduced numerous interventions prior, which were aligned with Geriatric Emergency Department Accreditation (GEDA) and resulted in being the first Level 1 site outside the United States. Efforts began in 2008, when raising awareness of specific risks of older adults, such as serious disease presenting with nonspecific symptoms, as well as undertriage of older patients.[10–13] Between 2011 and 2015, the modified Confusion Assessment Method for the Emergency Department (mCAM-ED), a delirium screening tool, was developed, implemented, and validated.[14,15] During the same period, team triage was introduced, incorporating a triage liaison physician to perform brief assessments, initiate both diagnostics

and treatment, and provide information.[16] While interprofessional collaboration in triage needed to be negotiated, collective decision-making enhanced triage quality in older patients.[17] Since 2017, all of our emergency clinicians follow age-friendly standards.[18] This includes, for example, practices such as age-appropriate pain management and performing delirium screening.[15,19] To support consistent care, our standards are an evidence-based, symptom-oriented clinical tool with algorithmic overviews and are accessible online to support decision-making on clinical shifts.[18] In 2018, an aggregated vital sign score was implemented, which was then combined with the Clinical Frailty Scale (CFS) to detect "geriatric urgency" and thereby enhance risk stratification. It also served as a means to initiate split flow of frail older patients to an age-friendly area.[20–22] In 2020, we established a Geriatric Emergency Medicine Specialist (GEMS) team with geriatric expertise, alongside expanded interdisciplinary and interprofessional collaboration with allied health professionals. In line with the GEDA program, a physician and nursing champion guide the department's age-friendly interventions through leadership, emergency clinician education, and continued quality improvement research (see Figure 2). These age-friendly initiatives also align with the hospital's organizational vision, strategy, and principles, including person-centeredness. Person-centered practice focuses on viewing people as individuals, respecting their right of self-determination, building trust and understanding, and fostering healthy relationships among patients, their informal care partners, and clinicians. Along with a holistic approach to care, person-centered care manifests itself in clinicians working with the patient's values and beliefs, or in other words: what matters most to patients.[1,23,24]

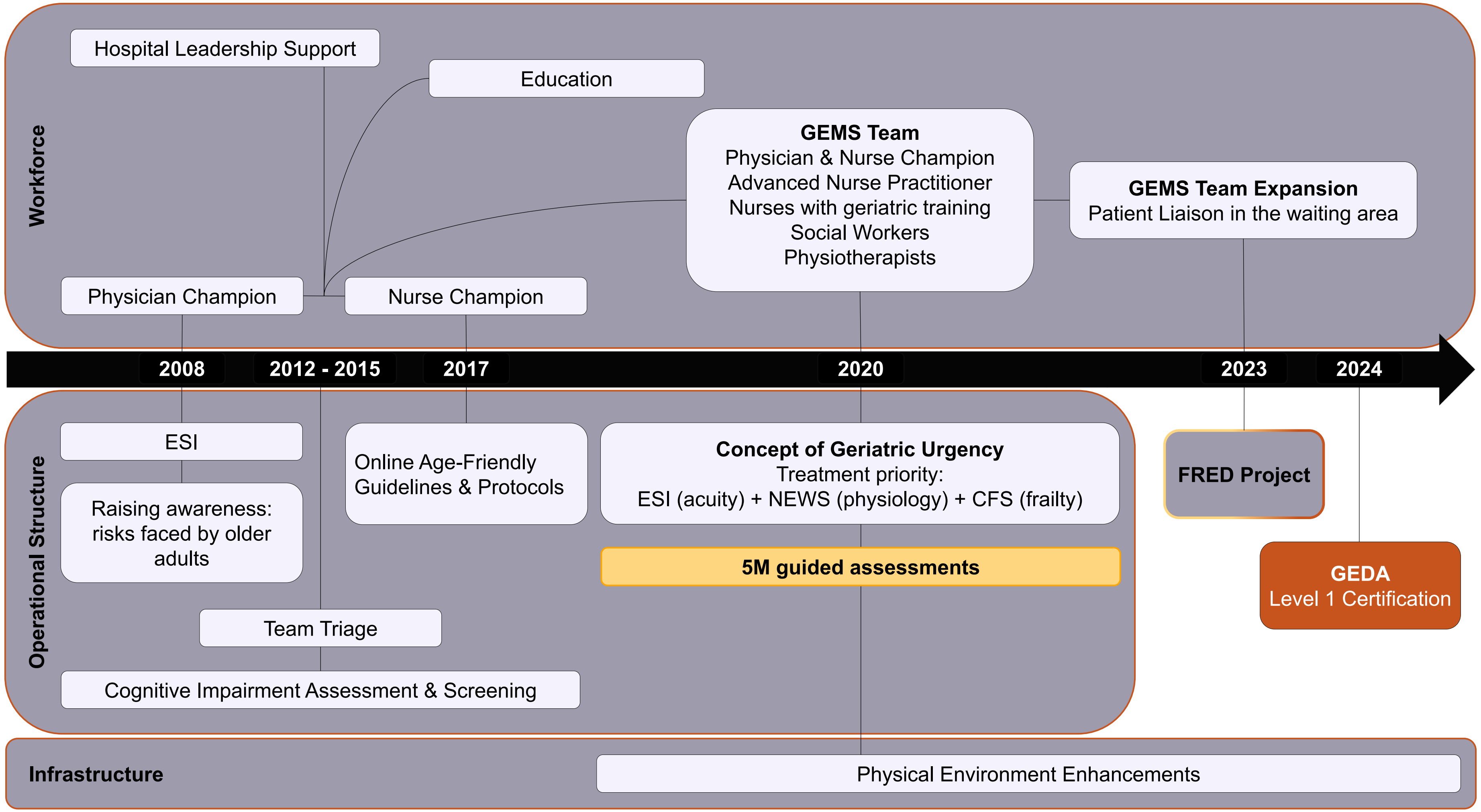

FIGURE 2. Overview of FRED's age-friendly intervention elements implemented at the University Hospital Basel, Northwestern Switzerland (2008–2024). The timeline shows how existing age-friendly practices evolved and were integrated into the age-friendly ED program developed

within the FRED project. In 2024, these efforts resulted in GEDA Level 1 accreditation, requiring verification of 20 care processes, quality improvement activities, and metrics tracking.[25]

GEMS, Geriatric Emergency Medicine Specialist; ESI, Emergency Severity Index;[26] NEWS, National Early Warning Score;[27] CFS, Clinical Frailty Scale;[20–22] 5M, Mind, Mobility, Multi-Complexity, Medications, Matters Most;[1] FRED, age-FRiendly Emergency Department; GEDA, Geriatric Emergency Department Accreditation.[28]

Our 20 GEDA Care Processes are:[25]

Baseline Care Processes Protocols: (1) minimize urinary catheter use; (2) minimize nil per os (NPO) status & promote access to food & drinks; (3) minimize use of physical restraints and promote use of trained companions or sitters instead.

Medication Safety and Orders Guidelines: (4) for minimize potentially inappropriate medication use; (5) for safe pain control.

Care Processes for ED Specialty Consultation Resources: (6) accessing palliative care consultation; (7) accessing geriatric psychiatry consultation; (8) guiding the use of volunteers in the care of older ED patients.

ED Screening Protocols with an Established Tool and Appropriate Follow-Up Actions: (9) structured delirium screening; (10) structured cognitive impairment screening; (11) structured assessment of function and functional decline; (12) structured falls and mobility assessment; (13) structured screening or assessment for elder abuse; (14) structured depression screening.

Care Processes for Transition of Care: (15) Primary Care Physician (PCP) notification of ED visit; (16) enabling transitions of care from the ED to residential care; (17) providing easy access to short- or long-term inpatient or outpatient rehabilitation services, and protocol or guidelines for how to access the pathway; (18) referrals to geriatric-specific follow-up clinics; (19) accessing an outreach program that provides home assessments of function and safety such as a visiting nurse association (VNA) or physical therapy (PT) home safety evaluation; (20) patient access to transportation services for return to their residence.

References Supplementary Text S1

1. Tinetti M, Huang A, Molnar F. The geriatrics 5m's: A new way of communicating what we do. *J Am Geriatr Soc*. 2017;65(9):2115. doi:10.1111/jgs.14979

2. American College of Emergency Physicians, American Geriatrics Society, Emergency Nurses Association, Society for Academic Emergency Medicine, Geriatric Emergency Department Guidelines Task Force. Geriatric emergency department guidelines. *Ann Emerg Med*. 2014;63(5):e7-25. doi:10.1016/j.annemergmed.2014.02.008

3. CMS Age Friendly Hospital Measure: Resources for Emergency Department. *Geriatric Emergency Department Collaborative (online)*. Published online August 28, 2024. Accessed June 25, 2025. https://gedcollaborative.com/resource/value-based-care/cms-hospital-measure-resources-for-eds/

4. Pelton LJ, Mate K, Snyder RE, Fulmer T. A path to better care for older adults: optimizing medicare and medicaid payments through implementation of the CMS age friendly hospital measure. *Inquiry*. 2025;62:469580251342460. doi:10.1177/00469580251342460

5. Conroy S, Nickel CH, Jónsdóttir AB, et al. The development of a European curriculum in Geriatric Emergency Medicine. *Eur Geriatr Med*. 2016;7(4):315-321. doi:10.1016/j.eurger.2016.03.011

6. Mooijaart SP, Nickel CH, Conroy SP, et al. A European Research Agenda for Geriatric Emergency Medicine: a modified Delphi study. *Eur Geriatr Med*. 2021;12(2):413-422. doi:10.1007/s41999-020-00426-8

7. Mooijaart SP, Lucke JA, Brabrand M, Conroy S, Nickel CH. Geriatric emergency medicine: time for a new approach on a European level. *Eur J Emerg Med*. 2019;26(2):75-76. doi:10.1097/MEJ.0000000000000594

8. Ellis B, Carpenter CR, Lowthian JA, Mooijaart SP, Nickel CH, Melady D. Statement on minimum standards for the care of older people in emergency departments by the geriatric emergency medicine special interest group of the international federation for emergency medicine. *CJEM*. 2018;20(3):368-369. doi:10.1017/cem.2017.426

9. Lucke JA, Mooijaart SP, Heeren P, et al. Providing care for older adults in the Emergency Department: expert clinical recommendations from the European Task Force on Geriatric Emergency Medicine. *Eur Geriatr Med*. 2022;13(2):309-317. doi:10.1007/s41999-021-00578-1

10. Nickel CH, Nemec M, Bingisser R. Weakness as presenting symptom in the emergency department. *Swiss Med Wkly*. 2009;139(17-18):271-272. doi:10.4414/smw.2009.12620

11. Nemec M, Koller MT, Nickel CH, et al. Patients presenting to the emergency department with non-specific complaints: the Basel Non-specific Complaints (BANC) study. *Acad Emerg Med*. 2010;17(3):284-292. doi:10.1111/j.1553-2712.2009.00658.x

12. Grossmann FF, Zumbrunn T, Frauchiger A, Delport K, Bingisser R, Nickel CH. At risk of undertriage? Testing the performance and accuracy of the emergency severity index in older emergency department patients. *Ann Emerg Med*. 2012;60(3):317-25.e3. doi:10.1016/j.annemergmed.2011.12.013

13. Grossmann FF, Zumbrunn T, Ciprian S, et al. Undertriage in older emergency department patients--tilting against windmills? *PLoS ONE*. 2014;9(8):e106203. doi:10.1371/journal.pone.0106203

14. Hasemann W, Grossmann FF, Stadler R, et al. Screening and detection of delirium in older ED patients: performance of the modified Confusion Assessment Method for the Emergency Department (mCAM-ED). A two-step tool. *Intern Emerg Med*. 2018;13(6):915-922. doi:10.1007/s11739-017-1781-y

15. Grossmann FF, Hasemann W, Graber A, Bingisser R, Kressig RW, Nickel CH. Screening, detection and management of delirium in the emergency department - a pilot study on the feasibility of a new algorithm for use in older emergency department patients: the modified Confusion Assessment Method for the Emergency Department (mCAM-ED). *Scand J Trauma Resusc Emerg Med*. 2014;22:19. doi:10.1186/1757-7241-22-19

16. Lauks J, Mramor B, Baumgartl K, Maier H, Nickel CH, Bingisser R. Medical team evaluation: effect on emergency department waiting time and length of stay. *PLoS ONE*. 2016;11(4):e0154372. doi:10.1371/journal.pone.0154372

17. Dreher-Hummel T, Nickel CH, Nicca D, Grossmann FF. The challenge of interprofessional collaboration in emergency department team triage - An interpretive description. *J Adv Nurs*. 2021;77(3):1368-1378. doi:10.1111/jan.14675

18. medStandards. *University Hospital Basel (online)*. Accessed July 29, 2025. https://medstandards.com/

19. Rat P, Jouve E, Pickering G, et al. Validation of an acute pain-behavior scale for older persons with inability to communicate verbally: Algoplus. *Eur J Pain*. 2011;15(2):198.e1-198.e10. doi:10.1016/j.ejpain.2010.06.012

20. Kabell Nissen S, Rueegg M, Carpenter CR, et al. Prognosis for older people at presentation to emergency department based on frailty and aggregated vital signs. *J Am Geriatr Soc*. 2023;71(4):1250-1258. doi:10.1111/jgs.18170

21. Kaeppeli T, Rueegg M, Dreher-Hummel T, et al. Validation of the Clinical Frailty Scale for Prediction of Thirty-Day Mortality in the Emergency Department. *Ann Emerg Med*. 2020;76(3):291-300. doi:10.1016/j.annemergmed.2020.03.028

22. Szczesna AZ, Nissen SK, Brabrand M, Bingisser R, Nickel CH. Validation of the frailty-adjusted prognosis tool for 30-day mortality in older emergency department patients. *Int J Clin Pract*. 2025;2025(1). doi:10.1155/ijcp/2048711

23. van den Ende ES, Schouten B, Kremers MNT, et al. Understanding what matters most to patients in acute care in seven countries, using the flash mob study design. *BMC Health Serv Res*. 2021;21(1):474. doi:10.1186/s12913-021-06459-4

24. McCormack B, McCance T, Bulley C, Brown D, McMillan A, Martin S. *Fundamentals of Person-Centred Healthcare Practice*. Hoboken, NJ: Wiley-Blackwell; 2021.

25. GEDA Care Processes Implementation and Tracking [PDF]. *American College of Emergency Physicians (online)*. Accessed July 4, 2025. https://www.acep.org/siteassets/sites/geda/media/documnets/geda-care-processes-and-evidence-of-implementation.pdf

26. Grossmann FF, Nickel CH, Christ M, Schneider K, Spirig R, Bingisser R. Transporting clinical tools to new settings: cultural adaptation and validation of the Emergency Severity Index in German. *Ann Emerg Med*. 2011;57(3):257-264. doi:10.1016/j.annemergmed.2010.07.021

27. Royal College of Physicians of London - Report of a working party. *National Early Warning Score (NEWS): Standardising the Assessment of Acute-Illness Severity in the NHS*. London: Royal College of Physicians; 2012.

28. Geriatric Emergency Department Criteria [PDF]. *American College of Emergency Physicians (online)*. Published online April 2024. Accessed June 25, 2025. https://www.acep.org/siteassets/sites/geda/media/documnets/geda-criteria.pdf

Supplementary Table S1: Manual and automatic extraction of variables and measurements from routine health data.

NR	Label	Values	Dependencies	Description	Note
**Manual extraction of variables based on Geriatric Emergency Department Accreditation (GEDA) care processes (#A-E)**					
**Record Identifications**					
1	Record ID	0001 to 1564 or<br>0001--1/2/3 to 1564--1/2/3		The Record ID, which must be entered manually in REDCap®. Entries from 0001 to 0210 and every 20th entry from 0210 onward were reviewed twice to assess interrater reliability. Consequently, some entries have suffixes --1 (Reviewer 1), --2 (Reviewer 2), and --3 (Consensus). Suffixes are for the Double Data Entry (DDE) function in REDCap®.	
2	Anonymous connection code	number between 1000000 and 1999999		The study code was generated by the hospital and consists of a randomized sequence from 0001 to 1564, combined with a randomized combination of the patient's date of birth.	Anonymous ID that can be traced back to the case ID (only on the hospital server trough the project leader ACF).
3	Reviewer	1= HL<br>2= WS<br>3= AME<br>4= consensus		Researcher who did the data reviewing.	
**Patient Identification and Triage**					
4	BS number	1= yes<br>2= no	If BS is '1= yes', the chart review ends here.<br>If BS is '2= no', further questions will appear.	Aims to determine whether a patient is identified with a "BS" number. These are patients with unknown identity (e.g., name, gender, date of birth), which leads to the exclusion for further review.<br>**Leading Question:**<br>Is the patient identified with a BS number?	If the reviewer identifies a BS number in the patient's case, the review will stop.<br>Otherwise, the review will continue with variable 5.
5	Trauma treatment	1= yes<br>2= no	If trauma is '1= yes', the chart review ends here.<br>If trauma is '2= no', further questions will appear.	Aims to determine whether a patient was treated in the resuscitation area, e.g., for major trauma or stroke. These patients are excluded from further review, regardless of the length of stay at the emergency department (ED).<br>**Leading question:**<br>Did the patient have a life-threatening situation and was treated in the resuscitation area?	If the reviewer identifies treatment in the resuscitation area in the patient's case, the review will stop.<br>Otherwise, the review will continue with variable 6.<br>ED = Emergency Department

Supplementary Table S1: Manual and automatic extraction of variables and measurements from routine health data.

NR	Label	Values	Dependencies	Description	Note
6	Presenting complaints	1 = none<br>2 = Fever<br>3 = Skin rash<br>4 = Headache<br>5 = Dizziness<br>6 = Acute visual disorder<br>7 = Acute hearing disorder<br>8 = Nasal discharge<br>9 = Dysphagia<br>10 = Cough<br>11 = Dyspnoea<br>12 = Chest pain<br>13 = Abdominal pain<br>14 = Nausea<br>15 = Vomiting<br>16 = Diarrhoea<br>17 = Obstipation<br>18 = Dysuria<br>19 = Back pain<br>20 = Neck pain<br>21 = Arm pain<br>22 = Leg pain<br>23 = Joint pain<br>24 = Flank pain<br>25 = Joint swelling<br>26 = Leg swelling<br>27 = Altered mental status<br>28 = Numbness<br>29 = Paralysis<br>30 = Gait disorder<br>31 = Speech disorder<br>32 = Fatigue<br>33 = Weakness<br>34 = Loss of appetite<br>35 = Sleeping disorder<br>36 = Spasms<br>37 = Agitation<br>38 = Suicidality<br>39 = Throat pain<br>40 = Hip pain<br>41 = Itching (pruritus)<br>42 = Urogenital complaints<br>43 = Comment (free text)		Aims to systematically determine which symptoms were present in patients at the time of their admission to the ED.<br>**Leading Question:**<br>At the time of presentation, which symptoms did the patient report experiencing? (Multiple answers allowed)	Multiple answers

Supplementary Table S1: Manual and automatic extraction of variables and measurements from routine health data.

NR	Label	Values	Dependencies	Description	Note
**Baseline care processes required by GEDA level 1 gold (#A1-3)**					
7	Urinary catheter (UC)	1= yes, new<br>2= yes, brought along<br>3= no	If UC '1= yes, new', Type of UC, Reason for UC, Provider, Timing and Details on the UC appear	Aims to determine whether a patient has a urinary catheter (UC). If so, a further distinction is made as to whether it was already in place before admission or was only inserted at the ED (new)?<br>**Leading question:**<br>Does the patient have a urinary catheter from the ED, from before ED presentation, or none?	GEDA:<br>**#A.1 Protocol or care process to standardize and minimize urinary catheter use.**<br><br>UC = urinary catheter
8	Type of urinary catheter (UC)	1= single use<br>2= IUC	If UC_type '2= IUC', Removing of the IUC at the ED appears	Aims to determine whether the UC used was a single-use catheter or an indwelling urinary catheter (IUC).<br>**Leading question:**<br>What type of urinary catheter did the healthcare worker use?	IUC = indwelling urinary catheter
9	Removing of the IUC at the ED	1= yes<br>2= no		Aims to determine whether the IUC was removed before the patient was discharged from the ED.<br>**Leading question:**<br>Was the IUC removed before discharge from the ED?	
10	Reason for UC	1. none<br>2. hourly urine output (include therapeutic consequence)<br>3. ESI 1<br>4. polytrauma<br>5. Ventilation, both non-invasive ventilation (NIV) or invasive<br>6. body surface area burn >20%<br>7. acute urinary retention<br>8. open sacral/perianal wound<br>9. trauma therapy requires immobilization (pelvic, spinal, hip fractures)<br>10. macrohematuria<br>11. perioperative<br>12. lower extremity trauma (UE) with immobilization<br>13. sacral/perianal macerations for incontinence, after all other measures for urinary drainage		Aims to indicate if a reason for inserting an UC is documented in the patient's health record.<br>**Leading question:**<br>If available, please provide the reason for the UC insertion. (Multiple answers allowed)	Multiple answers

Supplementary Table S1: Manual and automatic extraction of variables and measurements from routine health data.

NR	Label	Values	Dependencies	Description	Note
		14. palliative/comfort therapy at the request of the informed patient<br>15. urgent urine sample<br>16. others (free text)			
10a	Provider of UC	1= ED nurse<br>2= ED physician<br>3= consultation physician<br>4= others (free text)<br>5= unknown		Aims to determine who inserted the urinary catheter.<br>**Leading question:**<br>Who provided the insertion of a UC?	
10b	Timing of UC available	1= yes<br>2= no	If Timing of UC available '1= yes', then UC timing appears	Aims to determine if the timing of the urinary catheter insertion is available.<br>**Leading question:**<br>Is the date and time of the urinary catheter insertion recorded?	
10c	UC timing	DD-MM-YYYY HH:MM		Aims to determine when the insertion of a urinary catheter occurred.<br>**Leading question:**<br>When was the urinary catheter inserted? (date and time)	
10d	Details on the UC	free text		Aims to provide space for any additional details regarding the urinary catheter if identified during the chart review.<br>**Leading question:**<br>Remarks regarding the urinary catheter, if available.	
11	Nil Per Os (NPO) status	1= yes<br>2= no		Aims to determine if the patient had a nil per os (NPO) status prescribed by the physician or documented by the nurse in the health record.<br>**Leading question:**<br>Did the patient had nil per os status during the ED stay?	GEDA:<br>**#A.2 Protocol or care process to minimize NPO status and promote access to appropriate food and drink.**<br><br>NPO = nil per os (nothing by mouth)
12	Received food and/or drinks at the ED	1= yes<br>2= no	If Received food and/or drinks at the ED '1= yes', First food and/or drinks timing available appears	Aims to determine if the patient received food and/or drinks during their stay at the ED. **Leading question:**<br>Did the patient receive food and/or drinks during the ED stay?	
12a	First food and/or drinks timing available	1= yes<br>2= no	If First food and/or drinks timing available '1= yes', First food and/or drinks timing appears	Aims to determine if the timing of the first food/drink is available.<br>**Leading question:**<br>Is the date and time of the first food/drink recorded?	
12b	First food and/or drinks timing	DD-MM-YYYY HH:MM		Aims to determine how long the patient was without food and/or drinks.<br>**Leading question:**<br>When did the patient receive food and/or a drink for the first time at the ED? (date and time)	

Supplementary Table S1: Manual and automatic extraction of variables and measurements from routine health data.

NR	Label	Values	Dependencies	Description	Note
13	Use of trained companions or sitters	1= yes<br>2= no		Aims to indicate whether a one-to-one companion/ sitter (guard) was used instead of physical restraint.<br>**Leading question:**<br>Was there any use of a one-to-one sitter?	GEDA:<br>**#A.3 Protocol or policy to minimize use of physical restraints and promote use of trained companions or sitters instead.**
**Medication Safety and Orders (#B3)**					
14	Pain assessment	1= yes<br>2= no<br>3= impossible (reason: free text)	If Pain assessment '1= yes', Pain assessment timing available, Pain intensity, Pain reassessment and Pain management (intervention) appear	Aims to determine whether a pain assessment has been carried out. This is assessed regardless of the type of scale used.<br>**Leading question:**<br>Was a pain assessment conducted?	GEDA:<br>**#B.3 Guidelines for safe pain control including multi-modal options for mild, moderate, or severe pain.**
14a	Pain assessment timing available	1= yes<br>2= no	If Pain assessment timing available '1= yes', Timing of the assessment will appear.	Aims to determine if the timing of the pain assessment is available.<br>**Leading question:**<br>Is the date and time of the pain assessment recorded?	
14b	Timing of the assessment	DD-MM-YYYY HH:MM		Aims to determine the time and date of the initial pain assessment.<br>**Leading question:**<br>When was the pain assessment provided? (date and time)	
15	Pain intensity	1= NRS/VDS (free text, range 0-10)<br>2= text (free text)<br>3= Algo+ (free text, range 0-5)<br>4= others (free text)<br>5= impossible (free text)		Aims to determine the initial pain intensity of the patient.<br>**Leading question:**<br>What was the patient's initial pain intensity?	NRS = Numeric Rating Scale, VDS = Verbal Descriptor Scale
16	Pain reassessment	1= yes<br>2= no	If Pain reassessment '1= yes', Pain reassessment timing available, Pain reassessment intensity and Pain scale overall appear	Aims to determine if a reassessment of the pain level was conducted.<br>**Leading question:**<br>Was the pain reassessed?	

Supplementary Table S1: Manual and automatic extraction of variables and measurements from routine health data.

NR	Label	Values	Dependencies	Description	Note
16a	Pain reassessment timing available	1= yes<br>2= no	If Pain reassessment timing available '1= yes', Timing of the reassessment will appear.	Aims to determine if the timing of the pain reassessment is available.<br>**Leading question:**<br>Is the date and time of the pain reassessment recorded?	
16b	Timing of the reassessment	DD-MM-YYYY HH:MM	If the timing of the reassessment is provided, the 'Pain time difference' will be calculated automatically.	Aims to determine the time and date of the pain reassessment.<br>**Leading question:**<br>When was the pain reassessment provided? (date and time)	
17	Pain time difference	minutes		Automatic calculation of the time difference between the initial pain assessment and the reassessment.	
18	Pain reassessment intensity	1= NRS/VDS (free text, range 0-10)<br>2= text (free text)<br>3= Algo+ (free text, range 0-5)<br>4= others (free text)<br>5= impossible (free text)		Aims to determine the reassessed pain intensity.<br>**Leading question:**<br>What was the patient's reassessed pain intensity?	

Supplementary Table S1: Manual and automatic extraction of variables and measurements from routine health data.

NR	Label	Values	Dependencies	Description	Note
19	Pain management (intervention)	1= none<br>2= pain medication<br>3= splints<br>4= cooling (ice)<br>5= local anesthesia<br>6= patient declines intervention(s)<br>7= others (free text)	If '2= Pain medication', Pain medication levels according medStandard appears	Aims to determine the type of intervention conducted for pain management within one hour after the initial pain assessment.<br>**Leading question:**<br>Which pain management intervention was conducted within one hour? (Multiple answers allowed)	Multiple answers
20	Pain medication levels according to standard	1= Level 1<br>2= Level 2<br>3= Level 3<br>4= others (free text)<br>5= unknown		Aims to determine the level of medication administered for pain, according to the hospital's medStandards (levels 1-3).<br>**Leading question:**<br>What medication level (1-3) was administered?	MedStandards has been the knowledge management tool of the Department of Medicine and Acute Care at the University Hospital Basel since 2004. Age-friendly standards have been part of the ED's culture since 2017. They include practices like age-appropriate pain management. To support consistent care, these standards are evidence-based, symptom-oriented clinical tools with algorithmic overviews and are accessible online to support decision-making on clinical shifts. See on:<br>https://medstandards.com/medstandards-server/index?lang=EN#libraryItemId=18105242<br><br>Therapy Level 1: Basic analgesia<br>Therapy Level 2: Basic analgesia + opiate p.o.<br>Therapy Level 3: Basic analgesia + intravenous opiate
21	Pain scale overall	1= with NRS<br>2= with VDS<br>3= with Algo+<br>4= unstructured		Aims to determine which pain scale was utilized overall. In the case of recorded statements by the patients (e.g., "not so bad") or the use of different scales, "4 = unstructured" was used.<br>**Leading question:**<br>Which scale was used to measure the pain?	
**Specialty Consultant Resources (#C2)**					
22	Psychiatry consultation	1= yes<br>2= no		Aims to determine if a psychiatric consultant was involved in the ED care process.<br>**Leading question:**<br>Was a psychiatric consultant involved?	GEDA:<br>**#C.2 Care process for accessing geriatric psychiatry consultation in the ED.**

Supplementary Table S1: Manual and automatic extraction of variables and measurements from routine health data.

NR	Label	Values	Dependencies	Description	Note
**ED Screening (#D1-9)**					
23	Screening for cognitive impairment available	1= yes<br>2= no<br>3= impossible (reason: free text)	If Screening for cognitive impairment available '1= yes', 23 a-c/e, Result of the Month Backwards Test and mCAM-ED outcome appear	Aims to determine if a screening for cognitive impairment (e.g., Delirium Screening with the Month Backwards Test = MBT) was conducted during the ED care process.<br>**Leading question:**<br>Was a screening for cognitive impairment conducted, e.g., the MBT?	GEDA:<br>**#D.1 Protocol for structured delirium screening with an established tool, with appropriate follow-up actions based on screening results. Example tools include the DTS followed by the bCAM, 4AT, or others.**<br><br>MBT = Month Backwards Test
23a	How many mCAM-ED tools	exact number		Aims to identify the number of times the mCAM-ED tool was conducted on the patient during the ED stay.<br>**Leading question:**<br>How many times was the mCAM-ED tool conducted?	mCAM-ED = modified confusion assessment method for the emergency department<br>https://pubmed.ncbi.nlm.nih.gov/29290048/
23b	mCAM-ED provider	1= RN<br>2= GEMS nurse<br>3= unknown		Aims to determine who conducted the mCAM-ED, whether it was the RN, the nurse of the GEMS team, or an unknown individual.<br>**Leading question:**<br>Who conducted the mCAM-ED screening?	RN = registered nurse<br>GEMS = Geriatric Emergency Medicine Service
23c	mCAM-ED timing available	1= yes<br>2= no	If mCAM-ED timing available '1= yes', mCAM-ED timing appears	Aims to determine if the timing of the mCAM-ED is available.<br>**Leading question:**<br>Is the date and time of the mCAM-ED recorded?	
23d	mCAM-ED timing	DD-MM-YYYY HH:MM		Aims to determine the time when the mCAM-ED was conducted.<br>**Leading question:**<br>When was the mCAM-ED screening provided? (date and time)	
23e	Details on mCAM-ED	free text		Aims to provide space for any notable details related to the mCAM-ED in free text format.<br>**Leading question:**<br>Provide details on the intervention of the mCAM-ED if necessary.	
24	Result Month Backwards Test	1= pos<br>2= neg<br>3= impossible (reason: free text)	If Result Mont Backwards Test '1= pos', mCAM-ED evaluation appears	Aims to determine the result of the MBT. The results can be conspicuous (positive), non-conspicuous (negative), or not possible to conduct (which is counted as positive).<br>**Leading question:**<br>What is the result of the MBT?	
25	mCAM-ED evaluation	1= yes<br>2= no	If mCAM-ED evaluation '1= yes', mCAM-ED evaluation validation appears	Aims to determine the results of the mCAM-ED assessment. The assessment was only carried out if the MBT result was positive or not possible.<br>**Leading question:**<br>Did the healthcare worker document the result of the mCAM-ED assessment?	
26	mCAM-ED evaluation validation	1= yes<br>2= no		Aims to verify if the healthcare workers completed the delirium evaluation correctly according to the mCAM-ED.<br>**Leading question:**	

Supplementary Table S1: Manual and automatic extraction of variables and measurements from routine health data.

NR	Label	Values	Dependencies	Description	Note
				Did the healthcare worker fill in the result of the mCAM-ED correctly?	
27	mCAM-ED outcome	1= certain delirium<br>2= probably delirium<br>3 = no delirium		Aims to determine the result of the mCAM-ED assessment for the patient by distinguishing between delirium, probable delirium, or no delirium.<br>**Leading question:**<br>Regarding the mCAM-ED: What was the result of the screening and assessment?	
28	Delirium diagnosis mentioned	1= yes<br>2= no	If Delirium diagnosis mentioned '1= yes', Delirium diagnosis by name, place of documentation, medication mentioned and non-pharmacological interventions for patients with a delirium appear	Aims to determine whether the patient's chart states or indicates that the patient had delirium or a similar diagnosis, e.g., new confusion. This is independent of whether an mCAM-ED was conducted or not.<br>**Leading question:**<br>Was a diagnosis of delirium mentioned somewhere in the patient's chart?	
29	Delirium diagnosis by name	1 = Delirium<br>2 = (Unclear) confusion<br>3 = Encephalopathic condition<br>4 = Encephalopathy<br>5 = Altered mental status<br>6 = Decreased level of consciousness<br>7 = Impaired consciousness<br>8 = Alcohol withdrawal delirium (delirium tremens)<br>9 = Drug induced delirium<br>10 = others (free text)		Aims to determine if the patient's chart specifically states any of the following diagnoses: Delirium, (Unclear) confusion, Encephalopathic condition, Encephalopathy, altered mental status, decreased level of consciousness (or reduction of the GCS, or not A on AVPUC scale), impaired consciousness, alcohol withdrawal delirium (delirium tremens), drug - induced delirium, or any other relevant term.<br>**Leading Question:**<br>Which specific diagnosis related to delirium or altered mental status was mentioned in the patient chart? (Multiple answers allowed)	Multiple answers
30	Delirium place of documentation	1 = diagnosis list<br>2= To-do list<br>3= transfer or discharge report<br>4= procedure<br>5= progress (course)<br>6= others (Free text)		Aims to specify the location where the delirium diagnosis was documented. Answer options include the diagnosis list, the To-do list, the transfer or discharge report, procedure, or progress notes and others (free text).<br>**Leading question:**<br>Where was the diagnosis of the delirium documented? (Multiple answers allowed)	Multiple answers
31	Delirium medication mentioned	1= yes<br>2= no	If Delirium medication mentioned '1= yes', Delirium medication (32) and Delirium pharma medication dispensation (43) appear.	Aims to determine if any delirium corresponding medication was mentioned.<br>**Leading question:**<br>Is a drug therapy of delirium mentioned?	

Supplementary Table S1: Manual and automatic extraction of variables and measurements from routine health data.

NR	Label	Values	Dependencies	Description	Note
32	Delirium medication	1 = Quetiapin (Seroquel)<br>2= Haloperidol (Haldol)<br>3= Olanzapin (Zyprexa)<br>4= Risperidon (Risperdal)<br>5= Lorazepam (Temesta)<br>6= others (Free text)		Aims to specify the medication documented for delirium treatment. This includes both, fixed and reserve medications.<br>**Leading question:**<br>What specific delirium medication was documented for the patient? (Multiple answers allowed)	Multiple answers
33	Non-pharmacological interventions for patients with a delirium	1= yes<br>2= no	If non-pharmacological interventions for patients with delirium, '1= yes', Specification of non-pharmacological interventions for patients with a delirium appears	The aim is to determine the documentation of non-pharmacological interventions for delirium.<br>**Leading Question:**<br>Were non-pharmacological interventions for the treatment of delirium documented?	
34	Specification of non-pharmacological interventions for patients with delirium	**Caregiver Involvement & Support**<br>1 = involve relatives<br>2= private duty nurse or continuous care<br>3= team silver involved<br>4= counsellor involved<br>5= reference care (ideally only one nurse)<br><br>**Pain Management**<br>6 = pain treatment<br><br>**Patient mobility**<br>7= patient mobilized<br>8= patient seated in reclining chair (Lehnstuhl)<br><br>**Environment & Resources**<br>9= distraction (incl. newspapers, music)<br>10= placing patient in a quieter environment (incl. sector E)<br>11= orientation measures<br>12= hearing aids/visual aids<br>13= eating/drinking (not left on an empty stomach)<br><br>**Care Interventions**		The aim is to determine which specific non-pharmacological intervention was conducted at the ED.<br>**Leading question:**<br>What specific non-pharmacological interventions for the treatment of delirium were documented? (Multiple answers allowed)	Multiple answers

Supplementary Table S1: Manual and automatic extraction of variables and measurements from routine health data.

NR	Label	Values	Dependencies	Description	Note
		14= pay attention to urine output (incl. bladder scan)<br>15= remove unnecessary monitoring<br>16= remove or avoid use of infusion, bladder catheter incl. disposable catheter instead of permanent catheter<br><br>**Transfer Administration**<br>17= initiate rapid transfer			
35	Mental status	1= orientated<br>2= not orientated<br>3= new disorientated<br>4= unknown		Aims to determine the mental status of the patient, including whether the patient was oriented, not oriented, or newly disoriented.<br>**Leading question:**<br>What is the patient's mental status?	GEDA:<br>**#D2 Protocol for structured cognitive impairment screening with an established tool, with appropriate follow-up actions based on screening results. Tool: based on mCAM-ED and/or AMT-4**<br>Based on Tinetti M et al (2017), Grossmann F et al. (2014), Carpenter et al. (2019)
36	Mobility on admission ED (SUHB)	1= Stable gait<br>2= Unstable gait<br>3 = need Help to walk<br>4= Bedridden<br>5= assistive aids (free text)<br>6 = unknown		Aims to determine the patient's mobility at the time of admission to the ED according to the SUHB categorization.<br>**Leading question:**<br>What is the patient's mobility on admission to the ED?	GEDA:<br>**#D3 Protocol for structured assessment of function and functional decline with an established tool, with appropriate follow-up actions based on screening results. Tool: SUHB**<br>Kellet J. (2014)<br><br>SUHB Scale= Stable gait, Unstable gait, needs Help to walk, or Bedridden
37	Fall risk	1= yes<br>2= no		Aims to assess and address the fall risk of older patients. Emergency clinicians evaluate this through questioning, examinations, and observing the patient.<br>**Leading question:**<br>Was a fall risk assessment conducted and problems addressed?	GEDA:<br>**#D4 Protocol for structured falls and mobility assessment using an established tool, with appropriate follow-up actions based on screening results. Tool: STEADI**<br>Stevens JA et al. (2013)
38	Elder mistreatment	1= yes<br>2= no		Aims to determine if elder mistreatment at home is mentioned in the patient's chart. As there was no standardized or systematic recording of elder mistreatment at the time of data collection, this was most likely only recorded in cases where there was a suspicion of elder mistreatment.<br>**Leading question:**<br>Was elder mistreatment mentioned in the patient's chart?	GEDA:<br>**#D5 Protocol for structured screening or assessment for elder abuse using an established tool, with appropriate follow-up actions in response to screening results. Tool: ED Senior AID**<br>Platts-Mills TF et al. (2018)

Supplementary Table S1: Manual and automatic extraction of variables and measurements from routine health data.

NR	Label	Values	Dependencies	Description	Note
39	Depression screening	1= yes<br>2= no		Aims to determine if a depression screening was conducted during the patient's stay at the ED.<br>**Leading question:**<br>Was a depression screening conducted?	GEDA:<br>**#D6 Protocol for structured depression screening using an established tool, with appropriate follow-up actions in response to screening results. Tool: GDS short form**<br>Yesavage JA et al. (1982)
40	Social isolation screening	1= yes<br>2= no		Aims to determine if a social isolation was mentioned in the patient's chart. As with variable "Elder mistreatment", information on "Social isolation screening" was only recorded in those cases in which such a suspicion existed. E.g., statements such as "is socially well embedded" in the social anamnesis were generally not evaluated as "Social isolation screening". Social isolation must be explicitly documented in order for this to be recorded as a "yes".<br>**Leading question:**<br>Was a social isolation mentioned in the patient's chart?	GEDA:<br>**#D7 Protocol for structured screening or assessment for social isolation with appropriate follow-up actions in response to screening results. Example tools include the Duke Social Support Index and the UCLA 3-Item Loneliness Scale. ED Senior AID**<br>Platts-Mills TF et al. (2018)
41	Alcohol or substance use screening	1= yes<br>2= no		Aims to determin whether current alcohol or substance use was mentioned in the patient's chart.<br>**Leading question:**<br>Was an alcohol or substance use mentioned in the patient's chart?	GEDA:<br>**#D8 Protocol for screening for alcohol or substance use with appropriate follow-up actions in response to screening results. Tool: CAGE Questions (medStandards)**
42	Nutrition status	1= yes<br>2= no		Aims to determine if a nutrition status or screening was conducted during the patient's ED stay. The nutritional status of the patients was usually recorded based on their appearance. In this way, a distinction was made between e.g., cachectic, reduced, normal, overweight, and adipose nutritional status.<br>**Leading question:**<br>Was a nutrition screening conducted?	GEDA:<br>**#D9 Protocol for screening of nutritional status or food insecurity with appropriate follow-up actions in response to screening results. Tool: NRS.**
**ED screening (#D1) - Delirium Pharma (repeating instrument)**					
43	Delirium pharma medication dispensation	1= yes<br>2= no	If Delirium pharma medication dispensation '1= yes', Delirium pharma medication dispensation name, dosage and timing availability appear	Aims to determine if a delirium medication was dispensed to the patient.<br>**Leading Question:**<br>Was a delirium medication administered to the patient?	The dependency and continuation of variable 31.
44	Delirium pharma medication dispensation - name	1 = Quetiapin (Seroquel)<br>2= Haloperidol (Haldol)<br>3= Olanzapin (Zyprexa)<br>4= Risperidon (Risperdal)<br>5= Lorazepam (Temesta)<br>6= others (Free text)		Aims to determine the name of the medication that was dispensed to the patient.<br>**Leading Question:**<br>What was the name of the dispensed medication?	

Supplementary Table S1: Manual and automatic extraction of variables and measurements from routine health data.

NR	Label	Values	Dependencies	Description	Note
45	Delirium pharma medication dispensation - dosage	free text		Aims to determine the dosage of the medication that was dispensed to the patient.<br>**Leading Question:**<br>What was the administered medication dosage? (number and unit, with one space in-between)<br>If unknown, please indicate in the free text.	
46	Delirium pharma medication dispensation - timing availability	1= yes<br>2= no	If Delirium pharma medication dispensation timing availability '1= yes', Delirium pharma medication dispensation timing appears	The aim is to determine the availability of the time at which the medication was dispensed to the patient.<br>**Leading Question:**<br>Is the date and time of medication dispensing available?	
47	Delirium pharma medication dispensation - timing	DD-MM-YYYY HH:MM		Aims to determine the date and time at which the medication was dispensed to the patient.<br>**Leading Question:**<br>When was the medication dispensed to the patient? (date and time)	
**Transition of Care (#E5,7,10)**					
48	Geriatric-specific follow up: delirium unit	1= yes<br>2= no	If Geriatric specific follow up: delirium unit '2= no', Treated as outpatient appears	Aims to assess the care process for referrals to geriatric-specific follow-up clinics, i.e., delirium unit of the Department of Geriatric Medicine .<br>**Leading question:**<br>Has the patient been transferred to a specialized geriatric follow-up clinic (delirium unit)?	GEDA:<br>**#E.5 Care process for referrals to geriatric-specific follow-up clinics such as comprehensive geriatric care clinic, falls clinic, memory clinic, or others.**
49	Treated as outpatient	1= yes<br>2= no	If Treated as outpatient '1= yes', Care after discharging outpatients appears	Aims to determine whether the patient was treated as an outpatient during the treatment at the ED and was therefore subsequently discharged home.<br>**Leading question:**<br>Was the patient treated as an outpatient?	
50	Care after discharging outpatients	1= at home with new or additional home care services<br>2= no		Aims to assess whether additional professional care at home after discharge has been coordinated.<br>**Leading Question:**<br>Has there been any coordination of additional care at home, e.g., outpatient or home care services?	GEDA:<br>**#E.7 Care process for coordinating with a community paramedicine group to perform a home visit after discharge.**
51	Transport for return	1= yes<br>2= no		Aims to evaluate the patient's access to transportation services for their return to their residence.<br>**Leading question:**<br>Did the patient use a transportation service for their return to their residence?	GEDA:<br>**#E.10 Patient access to transportation services for return to their residence.**
**Others**					

Supplementary Table S1: Manual and automatic extraction of variables and measurements from routine health data.

NR	Label	Values	Dependencies	Description	Note
52	Note	free text		Anything that stood out and seemed to be important regarding a senior-friendly ED program.	
53	Time required for the review	minutes		How many minutes did the review process take for this case?	
**Automatic extraction of variables**					
54	Case Identification Number	00000000-99999999		Case ID of the patient	This study received an exemption from the Ethics Committee Northwest and Central Switzerland (Req-2023-01542). All procedures and data handling strictly adhere to the relevant ethical standards and legal regulations.
**Socio-demographic data**					
55	Gender	1= male<br>2= female		Sex of the individual. In the case of gender reassignment, the gender under civil law applicable at the time of admission to hospital must be stated.	
56	Age at entry	Numeric (exact age)		Age of the patient.	
57	Country of birth	text		Country in which the patient was born.	
58	Nationality	text		Nationality of the patient (name of the country).	
**Admission characteristics**					
59	Entry date and hour	DD-MM-YYYY<br>HH:MM:SS		Indication of the time of entry. For emergencies, the specification of the time is mandatory for emergencies.	
**Stay characteristics**					

Supplementary Table S1: Manual and automatic extraction of variables and measurements from routine health data.

NR	Label	Values	Dependencies	Description	Note
60	Type of treatment	1 = outpatient<br>2 = inpatient<br>3 = unknown		Specification of the treatment type	
61	Type of triage disposition	1= sector A with X-Ray<br>2= sector A without X-Ray<br>3 = sector B, C, D<br>4= sector E<br>5= GP emergency practice<br>6= medical polyclinic<br>7= others		After triage, which area was the patient referred to?	GP = General Practitioner
62	Transfer to internal to an ICU	DD-MM-YYYY<br>HH:MM:SS		Date and time if the patient was transferred from the ED to the ICU.	ICU = intensive care unit
63	Transfer to internal to an IMC	DD-MM-YYYY<br>HH:MM:SS		Date and time if the patient was transferred from the ED to the IMC.	IMC = intermediate care
**Discharge characteristics**					
64	Discharge date and hour	DD-MM-YYYY<br>HH:MM:SS		The time of discharge is optional. In the event of death, the exact date and hour of death is stated.	
65	Location after discharge	1= medical unit<br>2= surgical unit<br>3= acute geriatric care<br>4= others		Where was the inpatient discharged to?	
66	Discharge report	1= yes<br>2= no		Aims to determine if the physician has written a discharge report within 24 hours after the patient's stay in the ED.<br>**Leading question:**<br>Was the discharge report written within 24 hours?	GEDA:<br>**#E1 Care process for PCP (Primary Care Provider) notification of ED visit.**
**Diagnosis**					

Supplementary Table S1: Manual and automatic extraction of variables and measurements from routine health data.

NR	Label	Values	Dependencies	Description	Note
67	Main diagnosis	text		First main diagnosis from the Electronic Health Record at the ED.	
68	Main complaint	text		What was the chief complaint of the patient for this case ID?	
**Prediction models**					
69	Emergency Severity Index	numeric 1-5		A triage algorithm for assessing treatment severity.<br>**Leading question:**<br>What was the ESI (1-5)?	ESI = emergency severity index
70	National Early Warning System	numeric 0-20		An aggregated vital sign score serving as predictor tool for clinical deterioration.<br>**Leading question:**<br>What was the NEWS (0-20)?	NEWS = national early warning system
71	Clinical Frailty Scale	numeric 1-9		A scale used to assess vulnerability and to risk-stratify older ED patients.<br>**Leading question:**<br>What was the CFS score (1-9)?	CFS = clinical frailty scale

Supplementary Table S2: E-Survey questionnaire items

The statements were provided in German. Participants indicated their agreement on a 5-point Likert scale (strongly disagree, disagree, neutral, agree, strongly agree), except for the last item in the nurse questionnaire and the first item in the physician questionnaire, which were on own practices and job role:

**Nurse questionnaire**	**Physician questionnaire**
	Job role: Head of Department, deputy head, consultant physician, attending physician, resident physician.
There is good scientific evidence supporting the effectiveness of our delirium screening (mCAM-ED).	There is good scientific evidence supporting the effectiveness of our delirium screening (mCAM-ED).
Delirium screening should be implemented in all emergency departments for patients aged ≥ 65 years.	Delirium screening should be implemented in all emergency departments for patients aged ≥ 65 years.
The delirium screening tool is tailored to the needs of our emergency department.	The delirium screening tool is tailored to the needs of our emergency department.
The delirium screening tool is easy to understand and use.	
	The interpretation of the mCAM-ED is straightforward.
The delirium screening tool contributes to meeting patient needs and improving treatment.	The delirium screening tool contributes to meeting patient needs and improving care.

Nurse questionnaire	Physician questionnaire
Adequate delirium screening in the emergency department benefits the hospital and the healthcare system.	Adequate delirium screening in the emergency department benefits the hospital and the healthcare system.
The mCAM-ED screening tool we use is better suited than the tools used in other hospitals.	The mCAM-ED screening tool we use is better suited than the tools used in other hospitals.
Structural barriers (e.g., layout of the delirium screening form, workflow processes, resource allocation, organizational policies, technological limitations) in the emergency department affect the implementation of delirium screening.	
	The emergency department nursing staff is sufficiently trained to use the delirium screening tool.
	The treating team is aware of the risks of untreated delirium.
	The emergency department nursing staff conducts delirium screening on every patient aged ≥ 65 years.
There is a need for a reintroduction (re-implementation) of our delirium screening tool.	There is a need for a reintroduction (re-implementation) of our delirium screening tool.

**Nurse questionnaire**	**Physician questionnaire**
Delirium screening using the mCAM-ED is a high priority in the emergency department.	Delirium screening using the mCAM-ED is a high priority in the emergency department.
Our emergency department's resources are sufficient to conduct delirium screening as prescribed.	Our emergency department's resources are sufficient to conduct delirium screening as mandated.
Performing delirium screening has therapeutic consequences for patients.	Performing delirium screening has therapeutic consequences for patients.
Physicians review the delirium screening form.	
	As the responsible physician, I regularly review the delirium screening form.
I feel well-informed about recognizing and treating delirium.	I feel well-informed about recognizing and treating delirium.
A delirium screening tool, such as mCAM-ED, is necessary to diagnose delirium.	A delirium screening tool, such as mCAM-ED, is necessary to diagnose delirium.
I am familiar with the risk factors for developing delirium.	I am familiar with the risk factors for developing delirium.
I am familiar with the different forms of delirium.	I am familiar with the different types of delirium.
I am convinced of the effectiveness of the mCAM-ED tool in the early identification of delirium.	I am convinced of the effectiveness of the mCAM-ED tool in the early identification of delirium.

Nurse questionnaire	Physician questionnaire
I am confident in my ability to effectively use the mCAM-ED screening tool.	
My colleagues are confident in their ability to effectively use the mCAM-ED screening tool.	
	I am competent in interpreting the results of the mCAM-ED screening tool.
	My colleagues are competent in interpreting the results of the mCAM-ED screening tool.
	I trust that the emergency department staff uses and completes the delirium screening tool correctly.
I have observed resistance or skepticism among staff regarding delirium screening.	I have observed resistance or skepticism among staff regarding delirium screening.
I prefer caring for younger patients because they do not require delirium screening.	
The mCAM-ED screening tool fits well into the workflow of our emergency department.	
Competing demands or priorities in the emergency department make consistent delirium screening challenging.	Competing demands or priorities in the emergency department make consistent delirium screening challenging.
Training and support resources for using the mCAM-ED tool are readily available.	Training and support resources for using the mCAM-ED tool are readily available.

Nurse questionnaire	Physician questionnaire
I feel adequately trained and prepared to use the mCAM-ED screening tool.	
	I feel sufficiently trained and prepared to interpret the results of the mCAM-ED screening tool.
There are concerns about the acceptance of delirium screening by patients.	There are concerns about patient acceptance of delirium screening.
Please check all responses that represent barriers or prevent you from conducting delirium screening with the mCAM-ED for your older patients<br>Answer options:<br>▪ I always perform delirium screening on my older patients.<br>▪ It is not a priority<br>▪ I often forget<br>▪ I do not think it makes a difference<br>▪ I do not think it (mCAM-ED) is a good screening tool.<br>▪ It takes too long.	
